# Discovery of Novel Molecular Scaffolds to Overcome *Pseudomonas aeruginosa* Aminoglycoside Resistance: Insights for a Consensus Scoring Rational Design Approach

**DOI:** 10.3390/ijms27062642

**Published:** 2026-03-13

**Authors:** Francesco Iesce, Jochem Nelen, Alejandro Rodríguez-Martínez, Carlos Martínez-Cortés, Cristina Minnelli, Giovanna Mobbili, Alessandra Di Gregorio, Carla Vignaroli, Horacio Pérez-Sánchez, Roberta Galeazzi

**Affiliations:** 1 Department of Life and Environmental Sciences, Polytechnic University of Marche, via Brecce Bianche, 60131 Ancona, Italy; s1116398@studenti.univpm.it (F.I.); c.minnelli@staff.univpm.it (C.M.);; 2 Structural Bioinformatics and High Performance Computing Research Group (BIO-HPC), HiTech Innovation Hub, UCAM Universidad Católica de Murcia, 30107 Murcia, Spain

**Keywords:** *Pseudomonas aeruginosa*, multi-drug resistance, ligand-based virtual screening, efflux pump inhibitors, pharmacophore model, scaffold hopping

## Abstract

The berberine derivative 13-(2-methylbenzyl)-berberine (BED) has been shown to inhibit the MexXY-OprM efflux system of *Pseudomonas aeruginosa* (PA), a key contributor to aminoglycoside resistance, by interacting with the inner membrane protein MexY at an allosteric pocket (ALP). To enhance binding efficacy, this study aims to identify novel chemical scaffolds that target the MexY allosteric pocket through an integrated computational strategy. In this work, a ligand-based virtual screening (LBVS) approach was employed using a 2D/3D pharmacophore model derived from BED to perform in silico screening of an Enamine compound library, which encompasses a broad and diverse chemical space. A key objective was to compare the predictive performance of this pharmacophore-based workflow with a structure-based (SB) strategy incorporating molecular docking and molecular dynamics (MD) simulations. Notably, the top-ranked LBVS hits were consistently validated by docking and MD analyses, showing stable binding and interaction patterns comparable or superior to those of BED. This convergence between ligand-based (LB) and SB methods highlights the internal coherence of the workflow and supports the robustness of the pharmacophore hypothesis. The identified scaffolds generally displayed high hydrophobicity, consistent with the physicochemical nature of the binding site, but resulting in limited aqueous solubility and complicating their experimental evaluation. While these features confirm the importance of hydrophobic interactions in MexY recognition, with a particular focus on some few residues, such as Phe560, it also underscores the need for formulation strategies or rational scaffold modifications introducing moderate polarity without weakening key contacts. Overall, the integrated computational strategy not only yields promising lead chemical structures but also provides a solid basis for their future optimization, ultimately supporting the design of new efflux pump inhibitors (EPIs) capable of contributing to improved antibiotic susceptibility in multidrug-resistant PA strains.

## 1. Introduction

**Figure 1:**
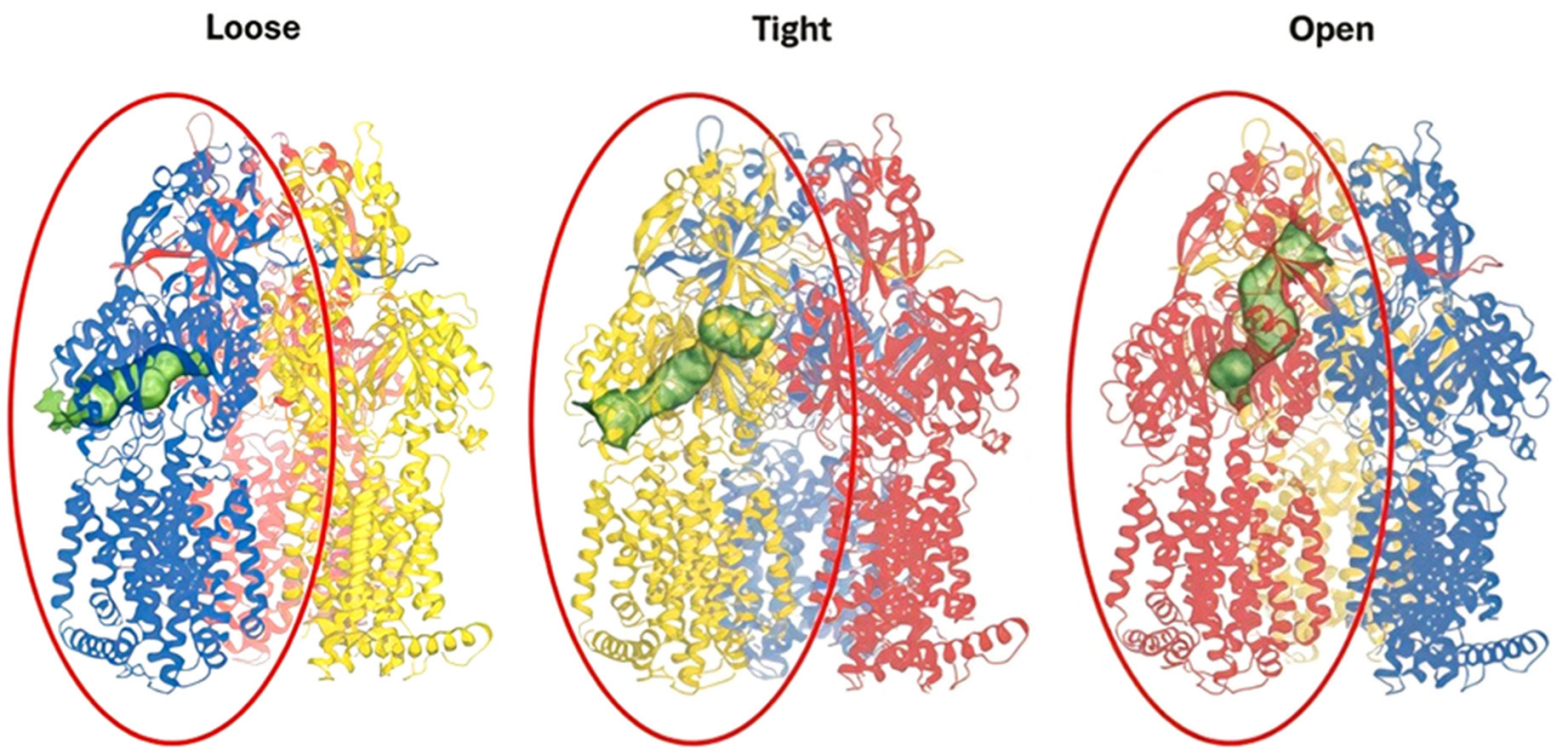
MexY Protein in trimeric LTO association. The monomer in Loose conformation is represented in blue ribbons, the Tight one in yellow and the Open conformation in red. The red circle highlights each of the three conformational states. Furthermore, the antibiotic access and extrusion channel is represented as a green volume on the surface maps, with a path that starts near the allosteric pocket and then goes outwards through the OprM channel.

**Figure 2:**
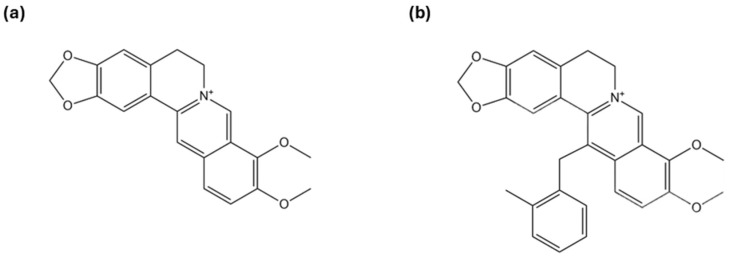
Representation of previous promising molecules. (**a**) 2D structure of the alkaloid berberine (BER); (**b**) 2D structure of BER’s most effective derivative 13-(2-methylbenzyl)-berberine (BED).

*Pseudomonas aeruginosa* is a Gram-negative bacterium that exhibits hallmark features of this group, including the presence of multidrug efflux pump (EP) systems [1,2] and a highly restrictive lipopolysaccharide (LPS)-rich outer membrane that severely limits drug entry. These intrinsic barriers, together with the downregulation of porin protein expression and the overexpression of efflux pumps (EPs), contribute substantially to antibiotic resistance [3,4]. Environmental selective pressure and horizontal gene transfer (HGT) are the primary drivers of the global spread of antibiotic resistance among Gram-negative bacteria [5]. In *P. aeruginosa*, the intrinsically low permeability of the cell envelope, combined with a wide range of intrinsic and acquired resistance mechanisms, markedly reduces the efficacy of many therapeutic agents [6]. Consequently, the treatment of chronic PA infections, such as those affecting the lungs of cystic fibrosis (CF) patients, remains a major clinical challenge [7]. This difficulty is largely attributable to the bacterium’s multi-drug resistant (MDR) phenotype and its remarkable ability to adapt to diverse antibiotic treatments [8,9]. In summary, infections caused by resistant PA strains continue to pose a significant global public health threat. It has been demonstrated that efflux pump expression is upregulated in *P. aeruginosa* during infection [10], driven by various stimuli such as oxidative stress [11]. To address this challenge, we focused on the MexXY-OprM efflux system as a promising therapeutic target, as it belongs to the resistance–nodulation–division (RND) family of transporters [12]. This multi-drug efflux system plays a crucial role in mediating resistance to aminoglycoside antibiotics [13] through their active extrusion from the bacterial cell [14]. Aminoglycosides exert their antibacterial activity by inhibiting bacterial protein synthesis, ultimately leading to reduced PA cell viability. Clinically, aminoglycosides are widely used for the treatment of severe *P. aeruginosa* infections, including chronic lung infections in patients with cystic fibrosis [7,10]. Tobramycin, in particular, is frequently administered by inhalation for the treatment of chronic PA lung infections in both cystic fibrosis and non-cystic fibrosis patients [10,15]. Notably, several studies have reported a high prevalence of multidrug-resistant PA strains overexpressing the *mexX*, *mexY* and *oprM* genes in pulmonary infections associated with cystic fibrosis [15]. Moreover, bacterial growth in biofilms in chronic *P. aeruginosa* infections, promotes the emergence of multiple mutant clones [16]. These clones frequently harbor inactivating mutations in *mexZ*, encoding for the transcriptional repressor of *mexXY* expression, resulting in constitutive overexpression of the MexXY-OprM efflux system and enhanced aminoglycoside resistance [10]. Consequently, targeting MexXY-OprM activity represents a compelling strategy to restore aminoglycoside efficacy, particularly in chronic and biofilm-associated infections where regulatory control of efflux pump expression is compromised. Several studies, including our own recent investigations [17,18,19], have highlighted the pivotal role of this efflux system in shaping the adaptive response of *P. aeruginosa* to antibiotic pressure. Thus, the modulation of efflux pump activity may restore bacterial susceptibility to conventional antibiotics, particularly when combined with adjuvant molecules (EPIs) capable of inhibiting expulsion function. Furthermore, from a structural point of view, other studies have elucidated the resistance mechanism mediated by the MexXY-OprM system. The EP is located in the periplasmic space of the Gram-negative PA cells and crosses the inner and outer membranes with its three components: MexY (inner part), MexX (periplasmic part) and OprM (outer part). Among these, MexY protein has been identified as the key determinant of substrate recognition and extrusion, owing to its strong homology with other substrate-binding RND transporters, including AcrB from *Escherichia coli* and related pumps, such as MexB, in *P. aeruginosa* [18,20,21]. Accordingly, MexY is proposed to operate via an extrusion mechanism analogous to that of AcrB [22,23], functioning through an asynchronous and asymmetric transport cycle that drives aminoglycoside expulsion from the bacterial cell [17]. Structurally, MexY assembles as a homotrimer in which each protomer sequentially adopts three distinct conformational states, Loose (L), Tight (T), and Open (O). These conformational transitions occur in a functional rotational manner and are powered by the proton motive force, collectively enabling efficient substrate transport across the cell envelope [22,23]. Drug binding occurs in the L state, after which the monomer adopts the T conformation to initiate expulsion. The process completes with the O conformation, through which the antibiotic is expelled from the cell (Figure 1). Computational analyses have shown that only the MexY tight conformation allows access to an allosteric site (ALP), which can be recruited in drug recognition [17]. Previous experimental studies have shown that the berberine (BER), an alkaloid with antimicrobial, anti-inflammatory, and glucose-lowering activities found in various plants extracts (e.g., *Berberis vulgaris*), can synergize with tobramycin against PA by blocking its efflux through binding to MexY [17,18]. Tobramycin is a potent aminoglycoside antibiotic widely used to treat Gram-negative infections; when administered by inhalation, it limits systemic toxicity compared with parenteral aminoglycosides. Moreover, its proven accumulation in the periplasmic space of *P. aeruginosa* enhances antibacterial efficacy and contributes to improved lung function, which is particularly relevant in respiratory diseases such as cystic fibrosis [24]. In this synergistic mechanism, berberine functions as an allosteric inhibitor of the MexY EP, thereby increasing intracellular antibiotic retention, allowing the tobramycin to exert its bactericidal activity by inhibiting protein synthesis. To further enhance BER’s efficacy, several synthetic berberine derivatives have been designed and evaluated against PA cell cultures [19,25]. Among these, 13-(2-methylbenzyl)-berberine (BED) emerged as one of the most promising candidates, exhibiting higher potency and improved activity compared to the parent compound berberine (Figure 2, Table 1) [19,26]. However, despite its enhanced efflux-inhibitory effect, BED does not fully restore PA susceptibility to tobramycin, indicating that inhibition of the MexXY-OprM system remains incomplete. Moreover, BED retains the rigid berberine scaffold, which may limit further optimization in terms of target engagement, chemical diversity, and physicochemical properties relevant to Gram-negative bacterial permeability. These limitations underscore the need to explore alternative chemical scaffolds capable of more effectively targeting the MexY ALP. Accordingly, the present study aimed to identify novel chemotypes able to block aminoglycoside efflux in *P. aeruginosa* and enhance antibiotic effectiveness. To this end, an LB pharmacophore modeling approach was applied to large molecular libraries using BED as the reference compound, with the aim of enabling scaffold hopping beyond the BER core [27]. The discovery of new efflux pump inhibitors remains particularly challenging, as effective molecules must combine high affinity for key residues of the efflux machinery with physicochemical properties compatible with the Gram-negative bacterial environment. Given this complexity, the integration of ligand-based and structure-based computational approaches is especially valuable. Their complementary nature can enhance predictive reliability and increase the likelihood of identifying truly effective EPIs capable of improving antibiotic efficacy in *P. aeruginosa*. By leveraging ligand-based virtual screening (LBVS), molecular docking, and molecular dynamics (MD) simulations within a unified workflow, this study expands the accessible chemical space and provides a rational framework for identifying scaffolds capable of stably engaging the MexY allosteric pocket. This integrated in silico strategy establishes a robust foundation for subsequent optimization efforts, ultimately supporting the rational design of drug-like inhibitors with the potential to improve antibiotic efficacy against resistant PA strains.

## 2. Results and Discussion

Despite previous studies identifying active molecules against *Pseudomonas aeruginosa* [19,26], our objective was to expand the accessible chemical space and facilitate scaffold hopping to discover structurally diverse bioactive compounds. To achieve this, we employed an integrated *state-of-the-art* computational drug discovery workflow, combining ligand-based and structure-based approaches, thereby improving the chances of identifying novel, potent effectors capable of overcoming existing limitations in efflux pump inhibition. As a first step, pharmacophore-based high-throughput virtual screening was performed using a combination of two different modes (with and without exclusion spheres) [28]. In both cases, 13-(2-methylbenzyl)-berberine (BED) was selected as the starting query, as this showed the highest activity among berberine derivatives in a previous study [19]. This LB virtual screening approach enabled the rapid examination of large molecular libraries and the prioritization of compounds which reproduced the key pharmacophoric features of previous validated inhibitors. Compared with experimental high-throughput screening (HTS), computational virtual screening offers a more efficient and cost-effective strategy to rationally narrow down the number of candidates for subsequent experimental testing [29,30,31]. To further strengthen and validate the initial candidates found using this in silico protocol, the shortlisted compounds were subsequently examined using structure-based methods, namely molecular docking and unbiased atomistic molecular dynamics simulations. In this phase, the goal was to evaluate the ability of each ligand to bind the allosteric pocket of MexY by analyzing binding poses, key protein–ligand interactions, and binding energies. Since the ALP site plays a crucial role in aminoglycoside extrusion, as supported by previous in silico and in vitro studies [18], this structure-based evaluation was essential to determine whether the LBVS hits were indeed capable of engaging the target in a manner comparable to, or better than the reference compound BED [32], a similar approach was previously used by Krovat et al. (2005) [33]. The agreement between pharmacophore-based filtering and subsequent receptor-level energetic, and structural analysis supports the robustness of the multi-staged computational approach used in this work.

### 2.1. Ligand-Based VS Results

**Figure 3:**
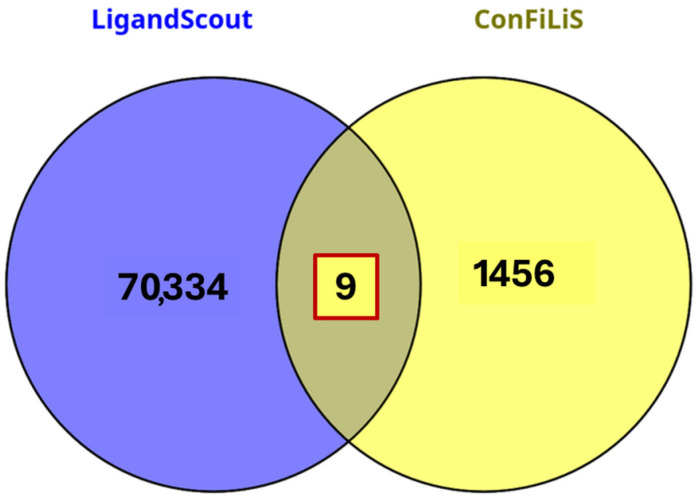
Display of the first mode LBVS results. Image by Venny web server showing the overlap between LigandScout results and ConFiLiS list of hit compounds by setting an *average consensus score* cut-off of −3.000.

**Figure 4:**
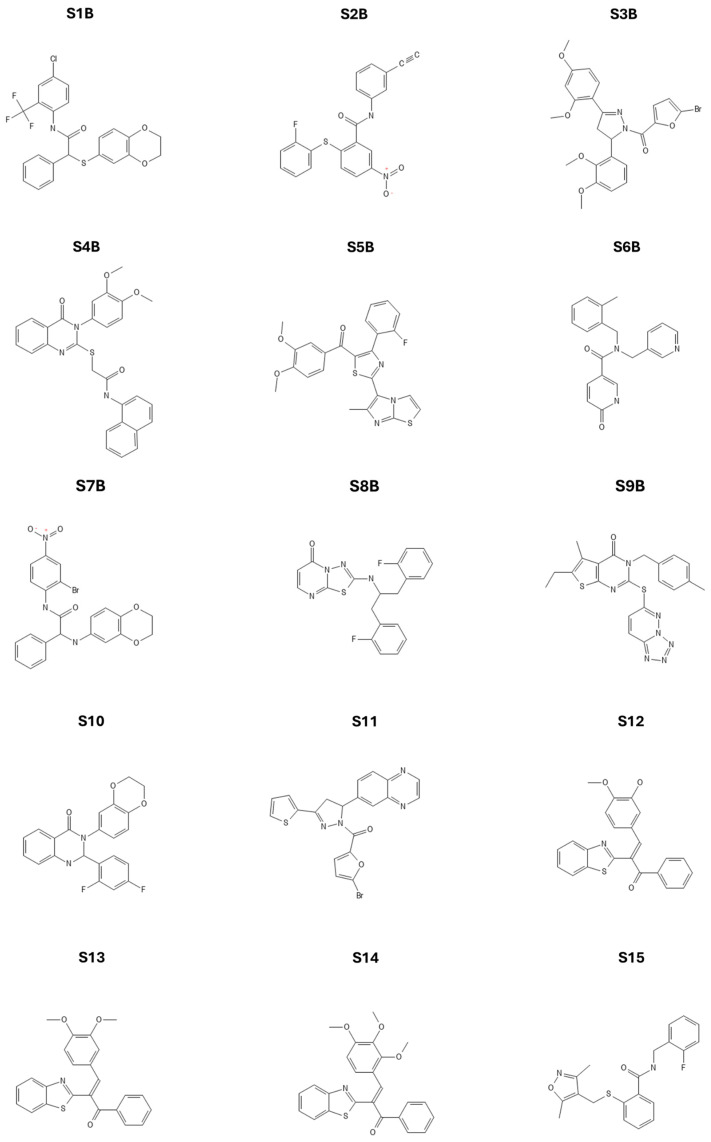

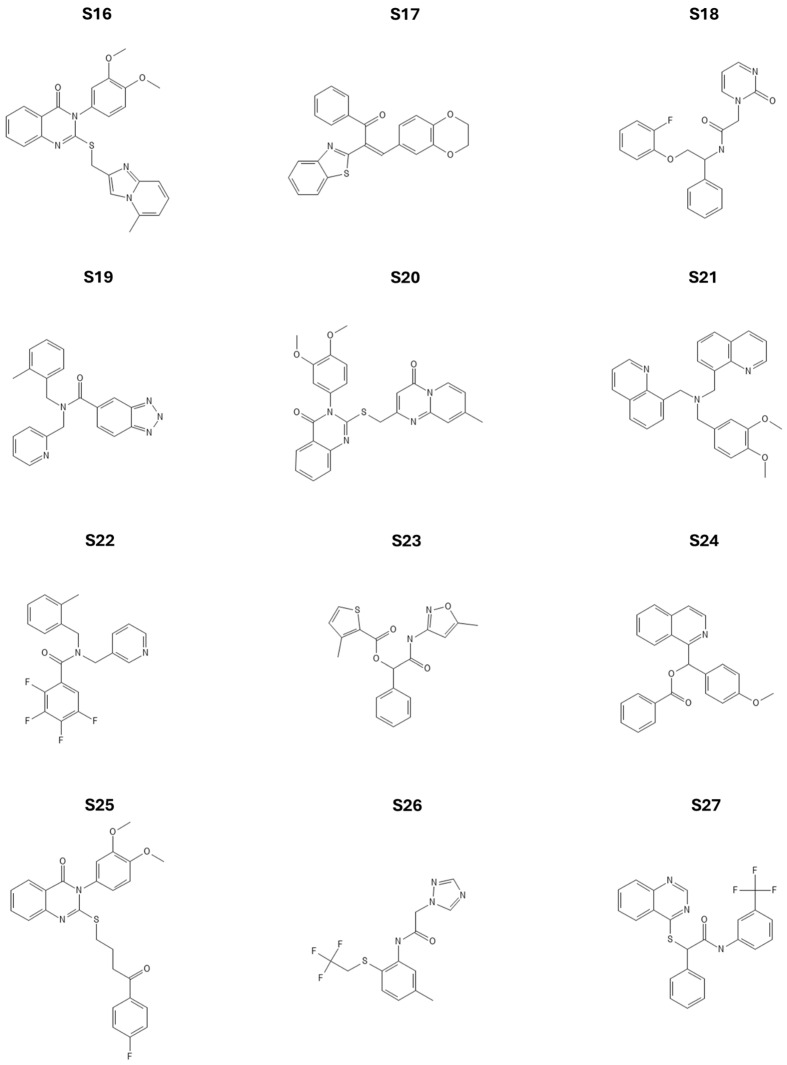
Chemical structures of lead compounds identified in the second LB virtual screening mode.

Pharmacophoric screening was central to filtering structurally diverse candidates based on BED-similarity with the aim of improving the potential EPIs activity against drug-resistant PA strains. Two modes of LB screening were performed. The first mode applied less stringent criteria, using a ligand-centric pharmacophore model derived from BED in which exclusion volumes were not included, and partial feature matching was allowed. In contrast, the second mode used a pharmacophore model derived from BED conformation resulting from bonding within the MexY allosteric pocket, incorporating only protein–ligand interaction-derived pharmacophore features as well as steric exclusion volumes. For this approach, full pharmacophore matching was required. Detailed settings and model construction and screening procedures are described in Section 3. Using the ENAMINE compound library and BED as query reference, the first screening mode yielded a large number of hits compounds (70,343). To further refine this set and prioritize molecules with higher predicted affinity to MexY, an additional filtering step was performed using ConFiLiS tool, which applies a consensus fingerprint similarity approach to rank compounds; further details are provided in Section 3. From its initial screening of the full ENAMINE library, ConFiLiS returned a ranked list based on consensus fingerprint similarity (2,899,338 compounds). Applying a threshold consensus score of −3.000 reduced this list to 1465 top candidates. By identifying compounds present in both the filtered ConFiLiS set and the LS-derived hits, 9 consensus molecules were obtained (Figure 3) and selected for further analysis given their chemical similarity to BED (Table 2). In the second screening mode, exclusion volume constraints were included to better match the shape of the ALP. This more selective approach yielded 27 hit compounds directly (Figure 4), without the need for further filtering or the step of molecular docking. Notably, one compound (L1B/S21: Z321002556) was identified by both screening modes, suggesting its potential as a strong EPI.

### 2.2. Molecular Docking of the First Mode Hits Compounds

**Figure 5:**
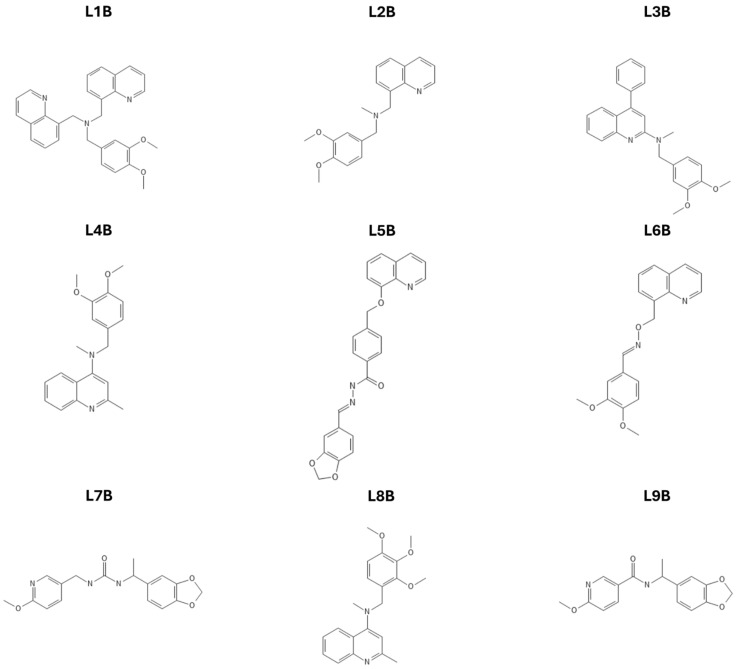
2D Chemical structures of lead compounds identified in the first LBVS mode.

To evaluate the binding affinity of the selected screening hits, molecular docking simulations were employed to explore ligand binding modes and identify the most favorable poses for subsequent refinement by atomistic molecular dynamics simulations. Docking results provided binding affinity estimates and structural insights into ligand recognition within the EP binding site, as obtained using the MGLTools/AutoDock4 platform. Since MexY is a strain-dependent polymorphic receptor, the MexY protein from the *Pseudomonas aeruginosa* PA7 strain (MexY^PA7^) was selected as the target for this study, based on the structural model previously optimized by Laudadio et al. (2019) [17]. The choice of the PA7 strain is particularly relevant, as it is characterized by elevated antibiotic resistance and overexpression of the MexXY–OprM efflux pump system, making it a suitable model for investigating inhibitor binding in a clinically challenging context. The inner membrane protein MexY adopts three distinct conformational states, namely tight (T), loose (L), and open (O). As highlighted in previous computational studies, the T conformation is the only state that presents potential allosteric binding sites capable of promoting allosteric inhibition of the protein. Consequently, this conformation was selected for docking calculations and all subsequent in silico investigations, and it was employed for all compounds reported in Figure 5. Using AutoDock4, the most favorable binding pose for each ligand was identified based on estimated binding enthalpy values. For each compound, the best-ranked docked cluster was selected (Table 3), and the corresponding protein–ligand complexes were then used as starting structures for the subsequent molecular dynamics simulations.

### 2.3. Molecular Dynamics Simulations Results

**Figure 6:**
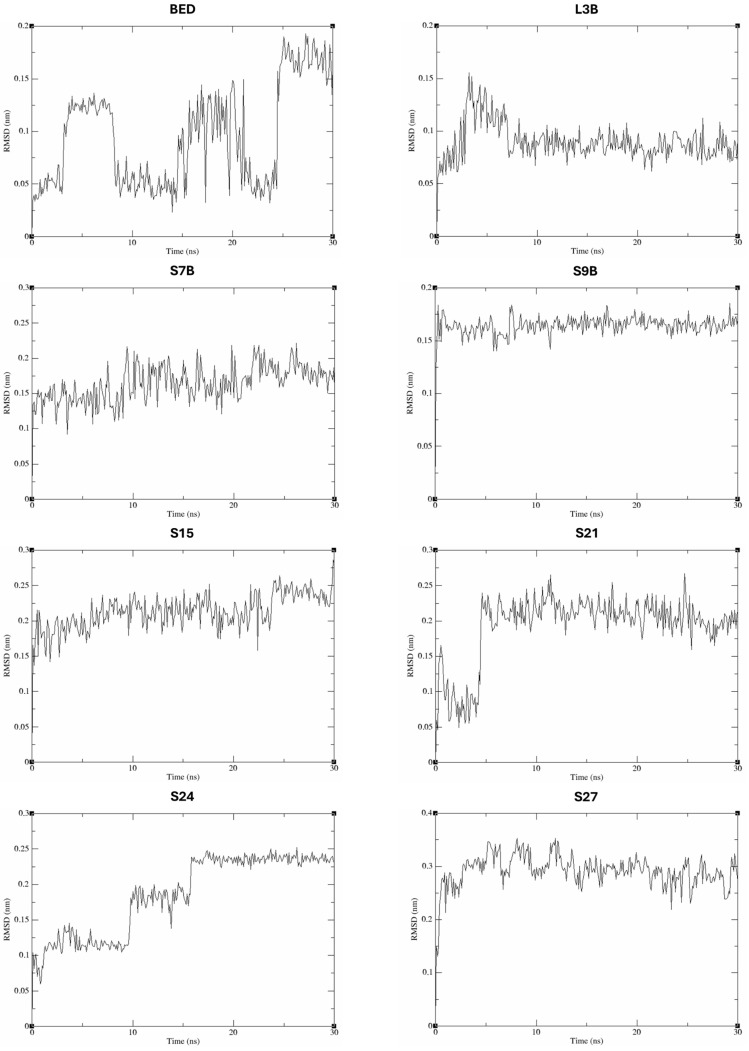
RMSD profiles of the selected lead compounds along the molecular dynamics trajectories. Time evolution (in nanoseconds, ns) of RMSD values for the selected ligands (L3B, S7B, S9B, S15, S21, S24, and S27) compared with the reference compound BED.

To assess the stability of ligand binding within the allosteric pocket (ALP), all protein–ligand complexes obtained from docking simulations (first mode), together with those in which the ligand was pre-docked (second mode), including the complex with the reference ligand BED, were subjected to unbiased molecular dynamics simulations. RMSD analyses indicated that all systems reached a stable conformational plateau within the first 10 ns of simulation, as shown in Figure 6. Data derived from the MD simulations, including RMSD, RMSF, and binding free energy estimations, were subsequently analyzed and filtered to identify the most promising lead candidates, which were then compared with the reference compound BED. Binding free energy calculations were performed using the MM-PBSA approach throughout the MD trajectories to estimate ligand binding affinities and to support the selection of the most promising candidates for further evaluation (Supplementary Tables S1 and S2 and Figure S1). Structural analyses of the stabilized complexes were carried out using UCSF ChimeraX (v1.10.1) and BIOVIA software (v2025) [34,35].

**Figure 7:**
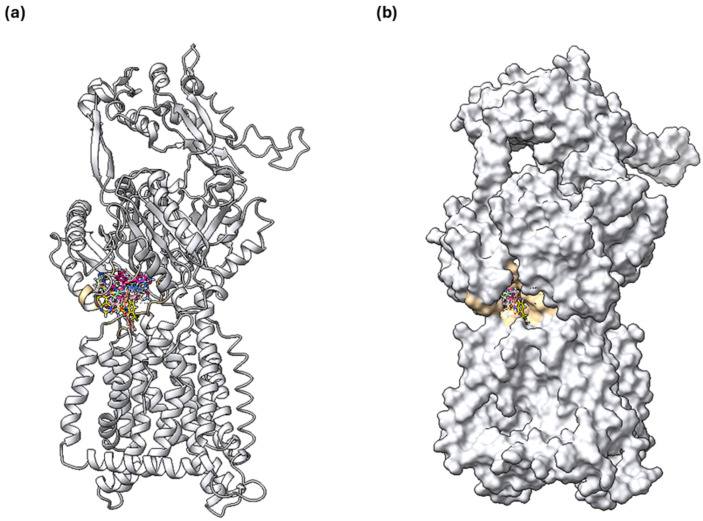
MexY tight (T) monomer showing the binding modes of the selected ligands within the allosteric pocket (ALP) compared to the reference ligand BED. Key ALP residues (Val462, Glu546, Gln558, Ala559, Phe560, Leu561, Pro562, Glu563, Pro664, Pro665, Leu666, Gly670, Ser671, Thr672, Ser673, Gly674, Phe675, Gln824, Ala825, Glu833, Ala834, Met835, Met838, Glu839, Trp853, Gln856, Ser857, Glu860, Arg861, Pro916) are shown in default colors. Ligands are shown as sticks: BED (default), L3B (blue), S7B (pink), S9B (light green), S15 (salmon), S21 (grey), S24 (magenta), S27 (gold). (**a**) Ribbon representation of the T conformer with docked ligands; (**b**) Solvent-accessible spheres representation of the T conformer with docked ligands.

**Figure 8:**
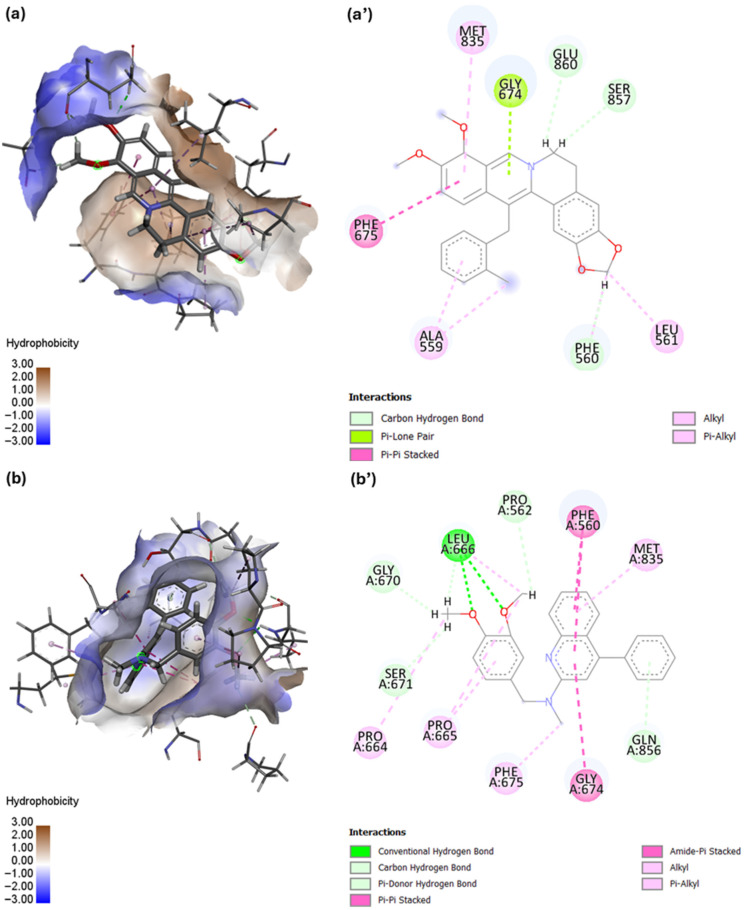

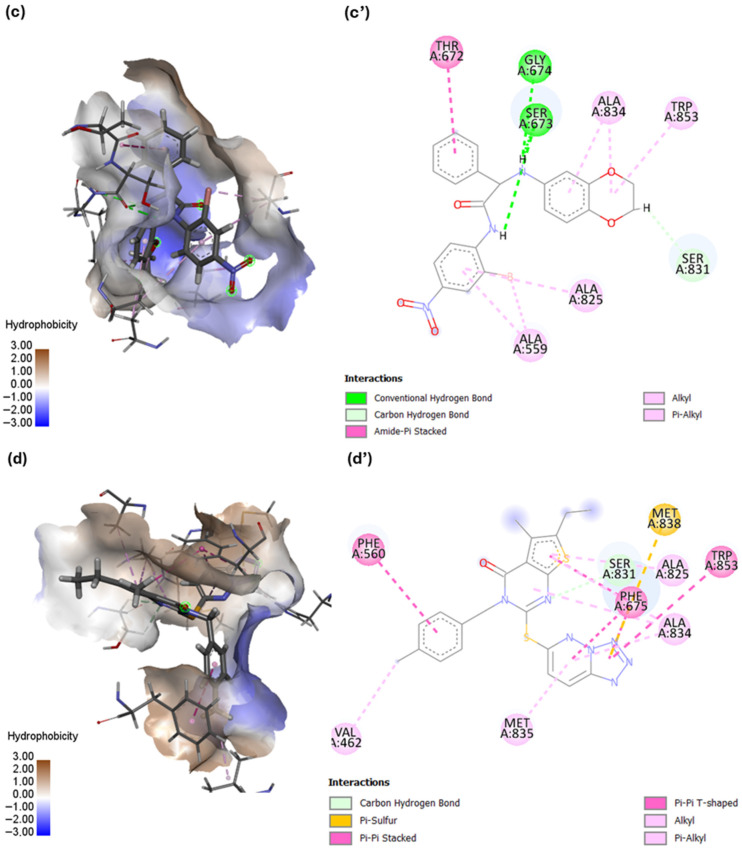

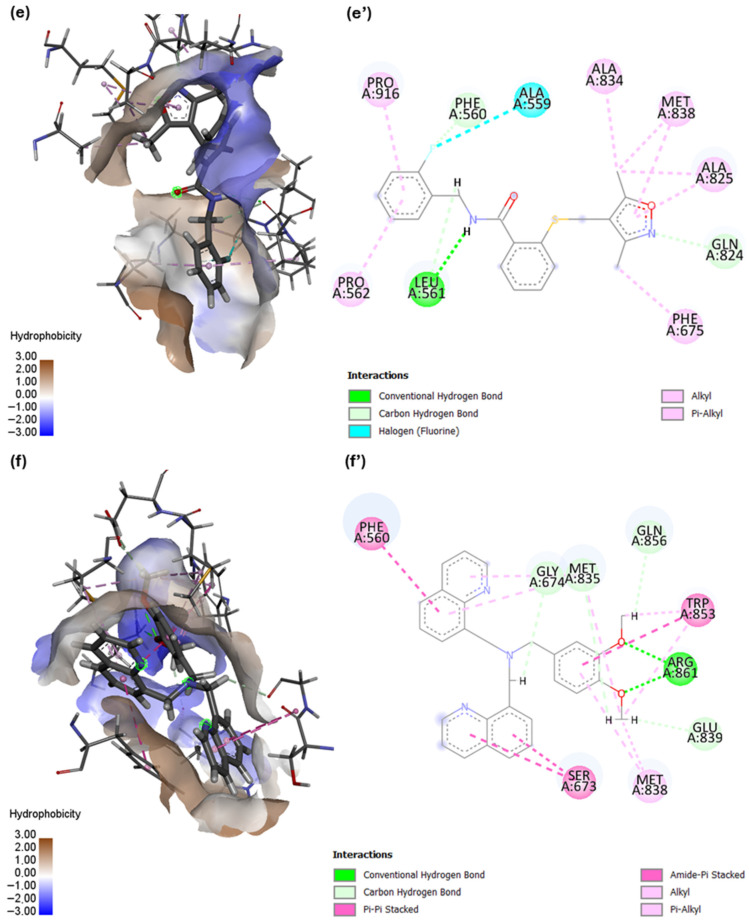

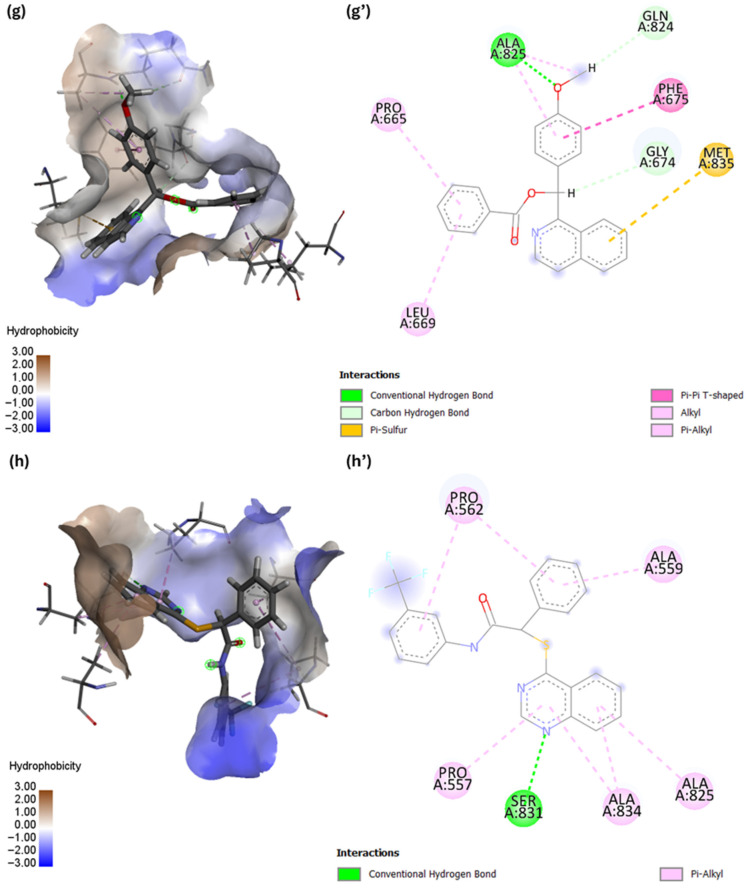
Representation of the selected ligands within the allosteric pocket (ALP) of the MexY tight monomer using BIOVIA Discovery Studio. (**a**–**h**) Stick models of ligands BED, L3B, S7B, S9B, S15, S21, S24, and S27, respectively, overlaid with their 3D hydrophobicity maps. The color gradient reflects the hydrophobicity of the ligand interaction surface, as indicated in the legend; (**a’**–**h’**) Corresponding two-dimensional interaction maps for the same set of ligands, illustrating key contacts within the ALP. The key interactions are shown in dashed lines colored as reported in the corresponding legend, which identify the nature of the contact.

All investigated compounds were found to fit properly and bind stably within the ALP, forming persistent interactions with multiple residues (Table 4, Figure 7), including key residues such as Phe560, Glu563, Ser673, Gln856 (Figure 8), which have previously been reported as critical for ligand recognition [36].

The reference ligand BED exhibited a ΔG_MM/PBSA_ value of −30.78 ± 3.42 kcal/mol. In the first binding mode, L3B showed a substantially more favorable binding free energy (−37.66 ± 3.89 kcal/mol). Even when considering the associated standard deviations, the difference between L3B and BED exceeds the combined uncertainty range, supporting a genuinely stronger predicted affinity. L1B (−29.70 ± 4.02 kcal/mol) displayed an energy fully comparable to BED within the error margins, whereas the remaining first-mode ligands (−26.51 to −17.44 kcal/mol) showed less favorable mean energies that, in most cases, fall outside BED’s uncertainty interval, suggesting weaker predicted binding. In the second binding mode, several compounds exhibited mean ΔG values close to that of BED, including S15 (−30.65 ± 3.40 kcal/mol) and S17 (−30.22 ± 3.25 kcal/mol), whose confidence intervals largely overlap with that of the reference ligand, indicating comparable stability. Notably, S21 (−38.17 ± 4.89 kcal/mol) and S9B (−35.29 ± 3.09 kcal/mol) showed more favorable mean binding energies than BED. For S9B, in particular, the difference relative to BED remains significant even when accounting for the standard deviations, whereas for S21 a partial overlap of the uncertainty ranges suggests a strong but more cautiously interpretable improvement. S7B (−33.71 ± 5.32 kcal/mol) also displayed a more favorable mean value, although the larger deviation warrants a more conservative interpretation of its energetic advantage. Overall, when the associated standard deviations are taken into account, several candidates demonstrate binding free energies that are either statistically comparable to or meaningfully more favorable than that of BED.

These findings further validate the robustness of the LBVS workflow in identifying promising hit compounds. Although minor differences were observed between the LBVS ranking and the subsequent docking and MM-PBSA results, all selected compounds displayed favorable (negative) binding free energies, confirming stable accommodation within the ALP binding pocket. The prioritization of a subset of candidates was primarily based on comparison with the reference ligand BED, which exhibited a particularly stable energetic profile and therefore represented a stringent benchmark. Compounds showing slightly weaker ΔG values were not excluded due to instability, but rather ranked lower relative to BED. These modest discrepancies can be rationalized by considering that the pharmacophore model employed during the LBVS stage was designed to capture the essential interaction features required for ligand recognition (e.g., hydrophobic/aromatic regions and hydrogen bond donor/acceptor features). While this ensures preservation of key binding determinants, dynamic and solvent-related contributions emerging during MD simulations may refine the energetic ranking, leading to small variations between virtual screening and post-MD evaluation. Thus, based on their energetic profiles, stability during MD simulations, and consistency of their binding modes within the ALP site, L3B, S7B, S9B, S15, S21, S24, and S27 were selected for further analysis and experimental evaluation (Figure 6).

### 2.4. Microbiological Preliminary Assays

On the basis of biological compound predictions, each selected molecule (L3B, S7B, S9B, S15, S21, S24, S27) was tested in microbiological assays (see methods in Supplementary Material). Owing to the intrinsic hydrophobicity of the compounds, only some molecules exhibited partial solubility in the culture medium, while others showed even more limited solubility. As a consequence, the maximum concentrations achievable in all microbiological assays were constrained by the upper DMSO threshold compatible with bacterial growth. This methodological limitation applies to all experiments described below and may have directly influenced the apparent lack or modest magnitude of the observed biological effects. The antibacterial activity of the compounds was first evaluated by MIC determination against the *P. aeruginosa* strains PA7 and PAO1. All compounds displayed MIC values > 320 µg/mL against both strains, indicating no evident intrinsic antibacterial activity under the tested conditions. Subsequently, checkerboard synergy assays were performed on the same strains by combining scalar concentrations of tobramycin with the compounds. Against the PAO1 strain, a one-fold reduction in the tobramycin MIC (from 0.5 to 0.25 µg/mL) was observed when combined with all compounds at any concentration tested (Supplementary Table S3). In contrast, against the PA7 strain, only compounds L3B, S15, S21, and S24 were able to reduce the tobramycin MIC from 256 to 128 µg/mL at concentrations ranging from 1.25 to 160 µg/mL (Supplementary Table S3). These results markedly differ from those previously reported [19] for the reference molecule BED, which produced a four-fold reduction in the tobramycin MIC in both strains: from 256 to 8 µg/mL in PA7 (at BED concentrations > 40 µg/mL) and from 0.5 to 0.06 µg/mL in PAO1 (at BED concentrations > 10 µg/mL).

Overall, the limited activity observed, particularly in the synergy assays, should be interpreted in light of the partial and variable solubility of the compounds and the associated DMSO constraints. Improving compound solubility will therefore be crucial for obtaining more potentially more informative results in future experiments.

### 2.5. Drug-like Property Predictions

In light of the limited and variable activity observed in the microbiological assays, in silico ADME-tox analyses (Absorption, Distribution, Metabolism, Excretion, and Toxicity) were performed to rationalize the experimental outcomes and to identify physicochemical factors potentially limiting the biological efficacy of the selected compounds. The ADME profiling of the seven compounds (L3B, S7B, S9B, S15, S21, S24, and S27), combined with their experimental physicochemical characterization, revealed a highly consistent profile characterized by pronounced lipophilicity (experimental LogP ≈ 4.6–6.1), moderate molecular weight (370–450 Da), and limited polarity (topological polar surface area, TPSA ≈ 47–55 Å^2^) predicted by ADME tools (Table 5, Supplementary Figure S2). While such properties may favor membrane association in eukaryotic systems, they are widely recognized as detrimental to efficient penetration across the outer membrane of Gram-negative bacteria. In particular, *Pseudomonas aeruginosa* possesses a highly impermeable, lipopolysaccharide-rich outer membrane and a restricted porin repertoire, which strongly disfavors the uptake of bulky and lipophilic molecules lacking sufficient polarity [37,38]. Accordingly, the predicted low aqueous solubility and high lipophilicity of the present compounds are expected to limit their diffusion through the outer membrane and reduce periplasmic and intracellular access. This permeability-limited phenotype is consistent with the absence of intrinsic antibacterial activity and with the modest effects observed in the synergy assays, especially considering the experimentally imposed constraints on maximum testable concentrations due to solubility and DMSO tolerance.

Within the series, a subset of compounds (e.g., S7B, S9B, and S15) exhibit relatively lower LogP values and higher TPSA compared to the others, indicating increased polarity. However, this increase was not sufficient to overcome the overall poor aqueous solubility, which limited their effective concentrations in microbiological assays (Supplementary Table S3) and likely contributed to the variability and modest magnitude of the observed biological effects. In addition to limited outer membrane permeation, the high LogP values likely hinder effective penetration through the bacterial envelope, ultimately restricting intracellular accumulation and facilitating active efflux. Lipophilic compounds, while well suited to engage the hydrophobic allosteric pocket (ALP) of MexY, are also preferential substrates of RND-type efflux pumps in *P. aeruginosa*. Therefore, active efflux is expected to further reduce intracellular exposure, reinforcing the permeability-driven limitations suggested by both ADME predictions and experimental data.

Despite these limitations related to Gram-negative penetration, the predicted safety-related ADME and toxicity parameters are overall favorable. None of the compounds displayed significant in silico alerts for mutagenicity (AMES test), carcinogenicity, or severe hepatotoxicity, and no critical hERG liability was predicted. Moreover, the absence of highly reactive functional groups and the moderate polar surface area suggest a low risk of nonspecific toxicity. Collectively, these findings indicate that, while optimization of physicochemical properties will be required to improve solubility and Gram-negative permeability, the current chemical series retains a promising safety profile suitable for further medicinal chemistry refinement.

## 3. Materials and Methods

### 3.1. Computational Methods

#### 3.1.1. Library Preparation

The chemical compounds library used for virtual screening (VS) was based on the Enamine High Throughput Screening (HTS) collection (https://enaminestore.com/), released in August 2021. This library includes over 2 million compounds and covers a broad chemical space, making it very suitable for the purpose of VS. To prepare the dataset for screening with LigandScout (LS) software (v4.4.7), the full library was converted into 217 LDB-format files using LigandScout’s included idbgen tool (available under the LigandScout license). Each file contained approximately 10,000 compounds (except for the last one), allowing for efficient parallelization and optimization of the screening process as demonstrated by Rodríguez et al., 2025 [40].

#### 3.1.2. Pharmacophore Model Generation

**Figure 9:**
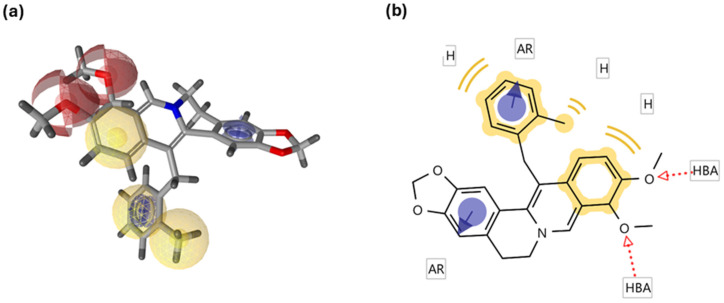
Representation of the generated pharmacophore. (**a**) 3D pharmacophore model of 13-(2-methylbenzyl)-berberine. (**b**) 2D model of the detailed possible interactions of the BED molecule: “H” (Hydrophobic), “AR” (Aromatic), “HBA” (Hydrogen bond acceptor).

Promising results from previous studies on BED compound [19,26] led to selecting it as the starting point for a pharmacophore-based virtual screening approach [29]. Its 3D pose and coordinates, obtained from prior computational work [19], was extracted and used as input for pharmacophore generation in LigandScout (v4.4.7) [28] (Figure 9). The software identified key pharmacophoric features (both 2D and 3D) such as hydrogen bond donors/acceptors, hydrophobic regions and aromatic rings, and combined them into a unified pharmacophore model. Two versions of the model were created: one including exclusion spheres and one without, the latter being more lenient in regard to matching the exact shape. The resulting models were saved in PMZ format and used directly for screening the Enamine HTS compound library.

#### 3.1.3. Ligand-Based Screening

**Scheme 1:**
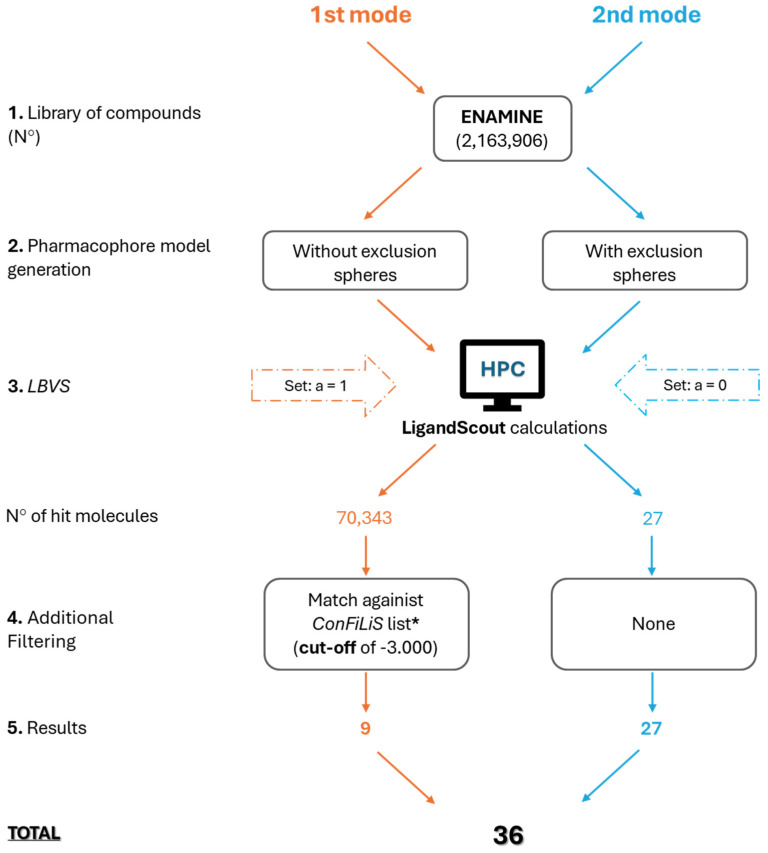
Workflow executed. Brief calculation summary showing a flowchart of both analysis modes performed, focusing on each step involving the screening for the hit molecules up to the final sets of drug-like compounds obtained. * ConFiLiS filtered hit compounds after application of the selected threshold.

The ligand-based virtual screening based on the modelled pharmacophores was performed using the iScreen module of LigandScout v4.4.7 [41], integrated within the MetaScreener software v.1.2 (https://github.com/bio-hpc/metascreener, accessed on 10 May 2025). Two slightly different workflows were followed: the first considering only the pharmacophore features of the ligand query itself (BED), while the second used the latter berberine derivative as a structural reference bound to the Tight monomer of MexY, reconstructed in silico by homology modelling [42], using the AcrB (*E. coli*) and MexB (*P. aeruginosa*) as templates [17,18]. In the first approach, all potential pharmacophore features of the ligand were considered independently of the target structure, while in the second one, the model was guided by the specific interactions formed within the protein binding site. The latter model was based on the allosteric pocket investigated in the previous articles (ALP) [17], in which the berberine and its derivatives were shown to bind with high affinity (Table 1). Given this, the pharmacophoric screening was carried out using Ligandscout’s iScreen module with different settings: in the first, the LigandScout flag “-a” (–allow_omit) was set to 1, defining the maximum number of pharmacophoric features from the query model that could be omitted during screening. This setting allowed partial matching of pharmacophoric features between the query compounds and the reference model, and was deliberately chosen as a looser criterion to accommodate the larger number of pharmacophore features included in this model. In addition, the PMZ-format pharmacophore file used in this mode did not include volume exclusion spheres. In this first screening mode, no additional physicochemical filters (e.g., Lipinski-based criteria) were applied prior to or after pharmacophore matching. Compound selection was exclusively based on pharmacophore similarity scoring within LigandScout, allowing omission of a maximum of one pharmacophoric feature (–allow_omit = 1) and without applying steric exclusion volumes. Therefore, the filtering criteria in this mode were strictly pharmacophore-driven. In the second configuration, the “-a” flag was set to 0, requiring full matching of all pharmacophoric features, and the PMZ file included steric volume exclusion, resulting in a more stringent selection of candidate compounds (Scheme 1).

#### 3.1.4. Selection of Hit Compounds

**Scheme 2:**
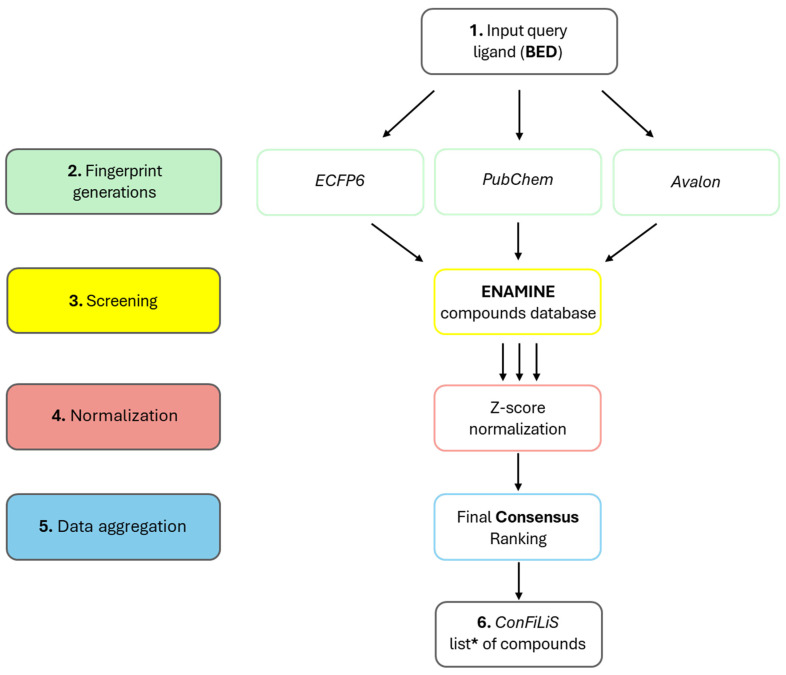
ConFiLiS calculations workflow. Summary of the main ConFiLiS workflow steps, starting from the input query molecule (BED), which is encoded using three different molecular fingerprints: *ECFP6*, *PubChem*, and *Avalon*. Each fingerprint representation is subsequently screened against the entirely Enamine compounds database. The resulting raw similarity data are subsequently normalized, and a final ranking is obtained based on the ConFiLiS *average consensus score*. * ConFiLiS filtered hit compounds after application of the selected threshold.

Compounds were selected based on their chemical similarity to BED, using the *pharmacophore similarity score* as calculated by LigandScout for both modes [28]. The first-mode workflow consisted of two independent filtering steps that were subsequently integrated. Initially, a pharmacophore-based screening was performed using LigandScout, generating a list of candidate compounds matching the BED-derived pharmacophore model. In parallel, the same chemical library was independently processed using ConFiLiS to rank compounds based on fingerprint similarity to BED. The final first-mode hit list was obtained by intersecting the top-ranking ConFiLiS compounds (after application of the −3.000 consensus score cut-off) with the LigandScout-derived pharmacophore hits. Indeed, due to the large number of predicted hit molecules obtained from the LS results of the first approach (70,343 hits), some additional filtering steps were performed. To refine the selection and narrow down the number of candidates, the ConFiLiS tool (v1.0, Consensus Fingerprints for ligand-based Screening, https://github.com/Jnelen/ConFiLiS, accessed on 10 May 2025), was used to generate an independent list of predicted similar compounds. First, ConFiLiS computed the molecular fingerprint of the query molecule (BED) using three different molecular fingerprints: *Avalon*, *PubChem*, and *ECFP6*. For each fingerprint representation, similarity between BED and library compounds was computed using the Tanimoto coefficient. The resulting similarity values were subsequently normalized within ConFiLiS using its built-in normalization procedure, and a final consensus score was obtained as the average of the three normalized fingerprint similarity scores. A consensus average score cut-off of −3.000 was then applied to retain the top-ranking candidates prior to overlap analysis with the LS-derived hit list. This configuration was adopted from a previous work, in which it enabled the successful identification of a curcumin derivative acting as an ABCC3 inhibitor [43]. Subsequently, it screened the fingerprints against the whole Enamine compounds database, achieving 2,899,338 compounds. The output was normalized using the built-in scoring function, and a consensus-based ranking was produced (Scheme 2). Thus, to reduce the size of the candidate pool, a consensus average score cut-off of −3.000 was applied to the ConFiLiS output, yielding 1465 top-ranking compounds. These compounds were then intersected with the 70,343 hits obtained from the first LigandScout pharmacophore-based screening. The overlap between the two independently generated hit lists was determined using the Venny tool (v2.1, https://bioinfogp.cnb.csic.es/tools/venny/index.html, accessed on 10 May–20 July 2025), resulting in 9 consensus compounds (Figure 4). These 9 molecules were retained as first-mode consensus hits and selected for further refinement through docking and molecular dynamics simulations.

In contrast, for the second screening run, a stricter LigandScout configuration was applied to improve shape complementarity and feature matching. The pharmacophore model included volume exclusion spheres to more accurately represent the ligand’s steric profile, and no pharmacophoric features were allowed to be omitted during matching (max omitted features = 0). This stricter LS screening yielded 27 hit compounds from the Enamine HTS library used. These molecules were retained for further analysis and subsequent refinement steps (Scheme 1). The original PubChem ID of each compound (https://pubchem.ncbi.nlm.nih.gov/) has been converted to an alias identifier; all of which are available into the Supplementary Materials (SM) Section (Tables S4 and S5).

#### 3.1.5. MexY Protein Preparation

The optimized 3D structure of the MexY trimer from the *Pseudomonas aeruginosa* 7 strain (PA7) was used for molecular docking and dynamics simulations [19,26,36] and it was built by homology modeling using MexB^PAO1^ (PDB ID: 2V50) as a template [21]. The model was energy-minimized in its biological environment (i.e., two leaflet POPC bilayer—168 lipids each) using CHARMM force field and CHARMM36m parametrization within GROMACS (v. 2024.3) framework [44], through a two-step protocol: 10,000 steps of steepest descent followed by 5000 steps of conjugate gradient minimization, until convergence (Fmax < 100 kJ/mol). The stabilized models were also compared to the recently published MexY^PAO1^ model (PDB ID: 9E9F) and confirmed the high predictability of the computational methods used (RMSD < 1 Å).

#### 3.1.6. Molecular Docking

The 9 compounds identified using the first LBVS approach were prepared for subsequent molecular docking. Firstly, we calculated the partial charges of each molecule by the AM1-BCC method within the UCSF Chimera (v.1.18) interface [45] and then saved as the corresponding mol2 files. These latter files were then uploaded as ligands in MGLTools and converted with full compatibility into the corresponding pdbqt format. Besides, the pdbqt file of the protein (MexY) was obtained by MGLTools/AutoDock4 (v.4.2.6) [46] software starting from the corresponding pdb coordinates. During the molecular docking experiments the receptor has been considered as rigid; the full flexibility will be considered during the subsequent molecular dynamics simulations. The selected molecules were docked in the ALP site of the MexY, performing a focus docking by the Lamarckian genetic algorithm (GA) with the x, y, z coordinates: 97.891, 77.896 and 110.846 respectively, involving the residues of the site able to establish interactions. The grid spacing was kept at 0.375 Å^3^, while the grid box dimensions were set to 70 × 70 × 70 Å^3^. The docking parameters were set to default values except for the number of GA runs, which was set to 50. The 27 compounds obtained from the second LBVS approach were already aligned to the docked BED molecule in the ALP site by LigandScout, and could therefore be used directly for subsequent MD simulations.

#### 3.1.7. Molecular Dynamics Simulations

The selected compounds by the first and second binding modes, previously docked to MexY, were further processed to generate starting structures for molecular dynamics (MD) simulations. Protein–ligand complexes were built using CHIMERA software v.1.18 [45] by combining the corresponding ligand mol2 files (calculated charges with AM1-BCC method) with the PDB structure of the MexY protein in tight (T) conformation. For comparison purposes, the MexY complex with reference compound BED was prepared following the same protocol. These complexes were then used as initial configurations for subsequent MD simulations. The simulations were performed with the ligands bound to MexY monomer (T) conformation, in an aqueous environment, as this setup was sufficient to evaluate binding stability and binding free energy for each potential lead compound in the relevant receptor conformation and enabled meaningful comparative analysis. The choice of a monomeric system in water was validated by comparison with the trimeric assembly embedded in a membrane, showing that the dynamics and ligand interactions within the ALP site are effectively captured by the simpler model. Indeed, the ALP binding site is relatively rigid and narrow, and all ligand–protein complexes reached equilibrium within 10–20 ns, indicating that longer simulations or the full trimer/membrane environment would not provide additional insights. Based on this analysis, we ended the MD trajectory to 30 ns, in order to consider a 10 ns range of full complex stabilization. This approach allows substantial savings in computational resources and data storage, while maintaining accuracy for comparative analysis across a large number of candidate compounds. The prepared protein-ligand complexes were subsequently submitted to the CHARMM-GUI web server [47] to generate the input files for MD simulations. The systems were solvated in explicit water using the TIP3P model (total number: 369,327 molecules) and supplemented with KCl to neutralize the system and achieve a physiological ionic strength of 0.15 M, corresponding to a total of 363 K^+^ and 351 Cl^−^ ions. Molecular dynamics simulations were performed using the CHARMM36m parametrization of the CHARMM force field by GROMACS 2024.3 on the high-performance DiSVA-HPC computing cluster [48]. Periodic boundary conditions (PBCs) were applied in all directions using a neighbor searching grid type (PBC dimensions: 160 × 160 × 160 Å^3^) and setting at 1.4 nm the cut-off distance for the short-range neighbor list. Electrostatic interactions were taken into account by implementing a fast and smooth Particle Mesh Ewald (PME) algorithm, with a 1.4 nm distance for the Coulomb cut-off [49]. Each simulation consisted of an initial energy minimization step, followed by equilibration in the NVT thermodynamic ensemble, and a 30 ns production in the NPT ensemble. This simulation time was chosen on the basis of previous published results on other BER derivatives [19,26], and was sufficient to achieve ligand stabilization within the ALP site, while not long enough to induce large-scale conformational rearrangements of the protein, as confirmed by backbone RMSD analyses (average RMSD 0.1–0.15 for all the simulated systems). A time step of 0.002 ps was used, with coordinates written out every 10 ps, and energy data collected every 2 ps. The temperature was set to 303.15 K using the V-rescale thermostat.

No positional or harmonic restraints were applied to the protein atoms during the production phase, allowing full relaxation of both ligand and receptor within the simulated timescale.

The MD simulations were carried out in duplicate to ensure statistical reliability. The strong agreement observed between the two independent trajectories likely reflects the small size of the binding site, which limits ligand mobility and reduces conformational variability.

The post-MD analyses were carried out using GROMACS tools (v.2024.3). Root mean square deviation (RMSD) graphs were generated for both protein and ligands, checking for stability. Also, RMSF of the whole protein and of the residues of the ALP site were monitored. Finally, we calculated the free binding energy for each ligand along the MD trajectory, by using the MM-PBSA method, in order to choose which compounds to test in vitro. The free Gibbs binding energy was calculated with the MM-PBSA method [50] (molecular mechanics/Poisson–Boltzmann surface area) using the g_mmpbsa tool (https://doi.org/10.1517/17460441.2015.1032936) [51]. MM-PBSA calculations were performed using the g_mmpbsa tool with its default parameters. In particular, polar solvation energies were computed using the Poisson–Boltzmann model, while non-polar contributions were estimated from the solvent-accessible surface area (SASA) term, according to the standard implementation of the tool. The ionic strength of the solvent was set to 0.150 M (saltcon = 0.150) to approximate physiological conditions. Default dielectric constants and grid settings were retained to ensure methodological consistency across all systems, as our objective was a comparative evaluation within the same receptor–ligand framework [52]. During the production run, snapshots were extracted every 10 ps for energy evaluation. Binding free energy values were calculated over the equilibrated portion of the trajectory, and mean ΔG values were obtained by averaging all sampled frames from the production run. The root mean square deviation was computed over all MD trajectories frames using GROMACS tools. Results are reported as mean values with standard deviations for all energetic components (Supplementary Tables S1 and S2 and Figure S1). The reported standard deviations were calculated over the full set of analyzed frames and therefore reflect the time-dependent fluctuation of ΔG along the MD trajectory. Time-dependent binding free energy profiles were also inspected to assess convergence and stability of the calculated values, and representative curves for the best-performing compounds are provided in Supplementary Figure S1.

#### 3.1.8. In Silico Prediction of ADME Properties

The ADMET properties of the experimental tested compounds were predicted using ADMET-AI [53], which applies a series of pre-trained machine learning models to estimate various pharmacokinetic and toxicity endpoints. The canonical SMILES of each compound was submitted to the web interface, and predictions were obtained for key parameters including human intestinal absorption (HIA), blood–brain barrier (BBB) permeability, cytochrome P450 (CYP450) inhibition, AMES toxicity for assessing the mutagenicity and potential carcinogenicity, and hepatotoxicity. Results were interpreted qualitatively and used to drive further optimization strategies.

## 4. Conclusions

The computational workflow developed in this study demonstrated strong internal consistency across the two ligand-based Virtual Screening runs. The pharmacophore models derived from the reference ligand 13-(2-methylbenzyl)-berberine (BED) successfully identified candidate molecules with novel chemical scaffolds, enabling effective exploration of the chemical space through scaffold hopping. Importantly, compounds prioritized during LBVS were consistently validated by molecular docking and MD simulations, which confirmed their ability to bind stably within the allosteric pocket of MexY (ALP).

In this context, the *consensus scoring* approach refers to the integrated prioritization of candidates based on the convergence of independent computational approaches, namely pharmacophore-based filtering (LigandScout/ConFiLiS) (LBVS), dynamic docking pose and scoring evaluation with subsequent MD/MM-PBSA energetic validation (SB). This multi-level agreement between ligand-based screening, structure-guided docking, and dynamic simulation strengthens the reliability of the pharmacophore hypothesis and highlights the robustness of the overall computational strategy. Compounds identified in the pharmacophore screening phase not only adopted favorable docking poses but also exhibited stable RMSD profiles and persistent interaction networks during MD simulations, demonstrating methodological coherence across complementary computational methodologies.

An additional insight from this workflow concerns the physicochemical properties of the selected scaffolds. Most top-ranked candidates display relatively high LogP values, reflecting the hydrophobic nature of the ALP environment, but this also results in limited aqueous solubility, which currently represents the main challenge for experimental evaluation. While this feature is consistent with the pocket’s characteristics, it complicates in vitro testing and handling. Furthermore, preliminary ADME evaluations suggest favorable pharmacokinetic profiles for the selected candidates, supporting their potential as drug-like molecules. Accordingly, future efforts will primarily focus on improving solubility through rational scaffold optimization, while preserving the key hydrophobic interactions required for allosteric binding.

Other developments will include experimental validation against a broader panel of *P. aeruginosa* clinical and reference strains to assess spectrum consistency, as well as structure–activity relationship (SAR) studies aimed at scaffold refinement and potency enhancement. In parallel, rational formulation strategies or suitable delivery systems could be explored to improve membrane permeability and intracellular accumulation, further enhancing the translational potential of these compounds. Overall, these results provide a solid foundation for the rational development of efficient EPIs and highlight solubility-driven optimization as a critical step toward translating the present computational findings into viable candidates against multidrug-resistant PA strains.

## Figures and Tables

**Table 1:** Docking-estimated Gibbs free binding energy values (ΔG_binding_, in kcal/mol) for berberine and its synthetic derivatives tested in silico against the MexY efflux transporter of *Pseudomonas aeruginosa* PAO1, PA7, and PA14 strains (data taken from the experimental work [19] on synergism with tobramycin). The red box indicates the best o-CH_3_ derivative (BED) result, and the strain for which it shows the highest affinity (PA7) is highlighted in bold. The 2D structures of all reported compounds can be found in reference [19].

Compound	ΔG_binding_ ( kcal/mol) MexY–PAO1	ΔG_binding_ ( kcal/mol) MexY–PA7	ΔG_binding_ ( kcal/mol) MexY–PA14
**Berberine**	−7.82	−8.66	−8.11
**o-CH_3_**	−8.54	**−10.56**	−9.38
**p-CH_3_**	−8.39	−10.48	−9.22
**p-CF_3_**	−9.03	−10.22	−9.28

**Table 2:** Best compounds from first-mode Ligand-based virtual screening analysis. Filtered results from the first screening approach, including LigandScout *pharmacophore similarity scores* and ConFiLiS *average consensus scores*.

Compound	Pharmacophore Similarity Score	ConFiLiS Score
**L1B**	0.955	−3.687
**L2B**	0.836	−3.597
**L3B**	0.835	−3.134
**L4B**	0.831	−3.280
**L5B**	0.830	−3.281
**L6B**	0.830	−3.055
**L7B**	0.829	−3.545
**L8B**	0.826	−3.063
**L9B**	0.824	−3.287

**Table 3:** Best binding poses for ligands selected from first-mode LBVS. Best-ranked binding pose for each first-mode selected hit compound, together with the corresponding docking-estimated Gibbs free binding energies (in kcal/mol).

Compound	Cluster Choice	ΔG_binding_ (kcal/mol)
**L1B**	1	−6.14
**L2B**	2	−5.44
**L3B**	1	−6.97
**L4B**	1	−5.29
**L5B**	1	−7.28
**L6B**	1	−6.03
**L7B**	2	−7.36
**L8B**	1	−5.59
**L9B**	1	−5.06

**Table 4:** ALP site interacting residues mapping.

Allosteric Pocket
Val462, Glu546, Gln558, Ala559, Phe560, Leu561, Pro562, Glu563, Pro664, Pro665, Leu666, Gly670, Ser671, Thr672, Ser673, Gly674, Phe675, Gln824, Ala825, Glu833, Ala834, Met835, Met838, Glu839, Trp853, Gln856, Ser857, Glu860, Arg861, Pro916

**Table 5:** Lipophilicity and binding affinity parameters of the tested compounds and the reference one BED. Experimental LogP values, total polar surface area (TPSA, in Å^2^), and estimated binding free energies (ΔG_binding_, in kcal/mol) are reported for each compound.

Compound	LogP (Exp) *	TPSA ** (Å^2^)	ΔG_binding_ (kcal/mol) ***	Solubility **	LogS
**L3B**	5.89	35	−37.66	3.40 × 10^−7^ mg/mL; 8.85 × 10^−10^ mol/L	−9.05
**S7B**	4.52	103	−33.71	1.12 × 10^−5^ mg/mL; 2.32 × 10^−8^ mol/L	−7.73
**S9B**	3.70	142	−35.29	2.58 × 10^−5^ mg/mL; 5.73 × 10^−8^ mol/L	−7.24
**S15**	3.86	80	−30.65	2.38 × 10^−6^ mg/mL; 6.41 × 10^−9^ mol/L	−8.19
**S21**	5.36	48	−38.17	8.59 × 10^−9^ mg/mL; 1.91 × 10^−11^ mol/L	−10.72
**S24**	5.33	48	−26.59	1.05 × 10^−6^ mg/mL; 2.84 × 10^−9^ mol/L	−8.55
**S27**	6.06	55	−21.09	2.24 × 10^−7^ mg/mL; 5.09 × 10^−10^ mol/L	−9.29
**BED**	0.43	40	−30.78	1.24 × 10^−2^ mg/mL; 2.76 × 10^−5^ mol/L	−4.56

* experimental LogP values reported in PubChem Database (https://pubchem.ncbi.nlm.nih.gov/). For BED, the LogP value has been obtained experimentally using the protocol described in reference [39] and reported in detail in the Supplementary Materials. ** calculated by SwissADME—SILICO-IT method—https://www.swissadme.ch/index.php (accessed on 10 November 2025). *** free Gibbs binding energy calculated by MM-PBSA method.

## Data Availability

The data presented in this study are available on request from the corresponding author due to privacy reasons.

## Supplementary Materials

The following supporting information can be downloaded at: https://www.mdpi.com/article/10.3390/ijms27062642/s1, Reference [54] is cited in the Supplementary Materials.

## References

[1] Nikaido H. (1996). Multidrug efflux pumps of gram-negative bacteria. J. Bacteriol..

[2] Piddock L.J.V. (2006). Multidrug-resistance efflux pumps? Not just for resistance. Nat. Rev. Microbiol..

[3] Webber M.A., Piddock L.J.V. (2003). The importance of efflux pumps in bacterial antibiotic resistance. J. Antimicrob. Chemother..

[4] Wu W., Huang J., Xu Z. (2024). Antibiotic influx and efflux in *Pseudomonas aeruginosa*: Regulation and therapeutic implications. Microb. Biotechnol..

[5] Wellington E.M.H., Boxall A.B.A., Cross P., Feil E.J., Gaze W.H., Hawkey P.M., Johnson-Rollings A.S., Jones D.L., Lee N.M., Otten W. (2013). The role of the natural environment in the emergence of antibiotic resistance in Gram-negative bacteria. Lancet Infect. Dis..

[6] Lambert P.A. (2002). Mechanisms of antibiotic resistance in *Pseudomonas aeruginosa*. J. R. Soc. Med..

[7] Poole K. (2005). Aminoglycoside Resistance in *Pseudomonas aeruginosa*. Antimicrob. Agents Chemother..

[8] Moradali M.F., Ghods S., Rehm B.H.A. (2017). *Pseudomonas aeruginosa* Lifestyle: A Paradigm for Adaptation, Survival, and Persistence. Front. Cell. Infect. Microbiol..

[9] Lister P.D., Wolter D.J., Hanson N.D. (2009). Antibacterial-Resistant *Pseudomonas aeruginosa*: Clinical Impact and Complex Regulation of Chromosomally Encoded Resistance Mechanisms. Clin. Microbiol. Rev..

[10] Aires J.R., Köhler T., Nikaido H., Plésiat P. (1999). Involvement of an Active Efflux System in the Natural Resistance of *Pseudomonas aeruginosa* to Aminoglycosides. Antimicrob. Agents Chemother..

[11] Fraud S., Poole K. (2011). Oxidative Stress Induction of the MexXY Multidrug Efflux Genes and Promotion of Aminoglycoside Resistance Development in *Pseudomonas aeruginosa*. Antimicrob. Agents Chemother..

[12] Morita Y., Tomida J., Kawamura Y. (2012). MexXY multidrug efflux system of *Pseudomonas aeruginosa*. Front. Microbiol..

[13] Hocquet D., Vogne C., El Garch F., Vejux A., Gotoh N., Lee A., Lomovskaya O., Plésiat P. (2003). MexXY-OprM efflux pump is necessary for an adaptive resistance of *Pseudomonas aeruginosa* to aminoglycosides. Antimicrob. Agents Chemother..

[14] Poole K. (2004). Efflux-mediated multiresistance in Gram-negative bacteria. Clin. Microbiol. Infect..

[15] Singh M., Yau Y.C.W., Wang S., Waters V., Kumar A. (2017). MexXY efflux pump overexpression and aminoglycoside resistance in cystic fibrosis isolates of *Pseudomonas aeruginosa* from chronic infections. Can. J. Microbiol..

[16] Sousa A.M., Pereira M.O. (2014). *Pseudomonas aeruginosa* Diversification during Infection Development in Cystic Fibrosis Lungs—A Review. Pathogens.

[17] Laudadio E., Cedraro N., Mangiaterra G., Citterio B., Mobbili G., Minnelli C., Bizzaro D., Biavasco F., Galeazzi R. (2019). Natural Alkaloid Berberine Activity against *Pseudomonas aeruginosa* MexXY-Mediated Aminoglycoside Resistance: In Silico and in Vitro Studies. J. Nat. Prod..

[18] Mangiaterra G., Cedraro N., Laudadio E., Minnelli C., Citterio B., Andreoni F., Mobbili G., Galeazzi R., Biavasco F. (2021). The Natural Alkaloid Berberine Can Reduce the Number of *Pseudomonas aeruginosa* Tolerant Cells. J. Nat. Prod..

[19] Giorgini G., Mangiaterra G., Cedraro N., Laudadio E., Sabbatini G., Cantarini M., Minnelli C., Mobbili G., Frangipani E., Biavasco F. (2021). Berberine Derivatives as *Pseudomonas aeruginosa* MexXY-OprM Inhibitors: Activity and In Silico Insights. Molecules.

[20] Lau C.H.-F., Hughes D., Poole K. (2014). MexY-promoted Aminoglycoside Resistance in *Pseudomonas aeruginosa*: Involvement of a Putative Proximal Binding Pocket in Aminoglycoside Recognition. mBio.

[21] Sennhauser G., Bukowska M.A., Briand C., Grütter M.G. (2009). Crystal Structure of the Multidrug Exporter MexB from *Pseudomonas aeruginosa*. J. Mol. Biol..

[22] Murakami S., Nakashima R., Yamashita E., Matsumoto T., Yamaguchi A. (2006). Crystal structures of a multidrug transporter reveal a functionally rotating mechanism. Nature.

[23] Seeger M.A., Schiefner A., Eicher T., Verrey F., Diederichs K., Pos K.M. (2006). Structural Asymmetry of AcrB trimer Suggests a Peristaltic Pump Mechanism. Science.

[24] Cheer S.M., Waugh J., Noble S. (2003). Inhaled Tobramycin (TOBI^®^). Drugs.

[25] Kavanaugh L.G., Mahoney A.R., Dey D., Wuest W.M., Conn J.L. (2023). Di-berberine conjugates as chemical probes of *Pseudomonas aeruginosa* MexXY-OprM efflux function and inhibition. npj Antimicrob. Resist..

[26] Giorgini G., Di Gregorio A., Mangiaterra G., Cedraro N., Minnelli C., Sabbatini G., Mobbili G., Simoni S., Vignaroli C., Galeazzi R. (2024). Inhibition of polymorphic MexXY-OprM efflux system in *Pseudomonas aeruginosa* clinical isolates by Berberine derivatives. ChemMedChem.

[27] Tanabe M., Sakate R., Nakabayashi J., Tsumura K., Ohira S., Iwato K., Kimura T. (2023). A novel in silico scaffold-hopping method for drug repositioning in rare and intractable diseases. Sci. Rep..

[28] Wolber G., Langer T. (2005). LigandScout: 3-D Pharmacophores Derived from Protein-Bound Ligands and Their Use as Virtual Screening Filters. J. Chem. Inf. Model..

[29] Wu H., Liu J., Zhang R., Lu Y., Cui G., Cui Z., Ding Y. (2024). A review of deep learning methods for ligand based drug virtual screening. Fundam. Res..

[30] McInnes C. (2007). Virtual screening strategies in drug discovery. Curr. Opin. Chem. Biol..

[31] Bajorath J. (2002). Integration of virtual and high-throughput screening. Nat. Rev. Drug Discov..

[32] Yang S.-Y. (2010). Pharmacophore modeling and applications in drug discovery: Challenges and recent advances. Drug Discov. Today.

[33] Krovat E.M., Frühwirth K.H., Langer T. (2005). Pharmacophore Identification, in Silico Screening, and Virtual Library Design for Inhibitors of the Human Factor Xa. J. Chem. Inf. Model..

[34] Pettersen E.F., Goddard T.D., Huang C.C., Meng E.C., Couch G.S., Croll T.I., Morris J.H., Ferrin T.E. (2021). UCSF ChimeraX: Structure visualization for researchers, educators, and developers. Protein Sci..

[35] BIOVIA, Dassault Systèmes (2025). BIOVIA Discovery Studio Visualizer.

[36] Tosiani V.D., Di Gregorio A., Giorgini G., Vignaroli C., Mari G., Mantellini F., Favi G., Minnelli C., Mobbili G., Simoni S. (2026). Stereochemical insight for MexXY-OprM efflux system inhibition in *Pseudomonas aeruginosa* from a pool of dihydro and tetrahydro berberine derivatives. Chem. Biol. Interact..

[37] Nikaido H. (1989). Outer membrane barrier as a mechanism of antimicrobial resistance. Antimicrob. Agents Chemother..

[38] Hancock R.E.W., Speert D.P. (2000). Antibiotic resistance in *Pseudomonas aeruginosa*: Mechanisms and impact on treatment. Drug Resist. Updates.

[39] Battu S.K., Repka M.A., Maddineni S., Chittiboyina A.G., Avery M.A., Majumdar S. (2010). Physicochemical Characterization of Berberine Chloride: A Perspective in the Development of a Solution Dosage Form for Oral Delivery. AAPS PharmSciTech.

[40] Rodríguez-Martínez A., Giraldo-Ruiz L., Ramos M.C., Luque I., Ribeiro D., Postigo-Corrales F., Alburquerque-González B., Montoro-García S., Arroyo-Rodríguez A.B., Conesa-Zamora P. (2025). Discovery of Z1362873773: A novel fascin inhibitor from a large chemical library for colorectal cancer. Sci. Rep..

[41] Langer T., Hoffmann R.D., Bachmair F., Begle S. (2000). Chemical function based pharmacophore models as suitable filters for virtual 3D-database screening. J. Mol. Struct. THEOCHEM.

[42] Hillish A., Pineda L.F., Hilgenfeld R. (2004). Utility of homology models in the drug discovery process. Drug Discov. Today.

[43] Nelen J., Naponelli V., Villalgordo-Soto J.M., Falasca M., Pérez-Sánchez H. (2025). Targeting Drug Resistance in Cancer: Dimethoxycurcumin as a Functional Antioxidant Targeting ABCC3. Antioxidants.

[44] Abraham M., Alekseenko A., Basov V., Bergh C., Briand E., Brown A., Doijade M., Fiorin G., Fleischmann S., Gorelov S. (2024). GROMACS 2024.3 Source Code.

[45] Pettersen E.F., Goddard T.D., Huang C.C., Couch G.S., Greenblatt D.M., Meng E.C., Ferrin T.E. (2004). UCSF Chimera—A visualization system for exploratory research and analysis. J. Comput. Chem..

[46] Morris G.M., Huey R., Lindstrom W., Sanner M.F., Belew R.K., Goodsell D.S., Olson A.J. (2009). AutoDock4 and AutoDockTools4: Automated docking with selective receptor flexibility. J. Comput. Chem..

[47] Jo S., Kim T., Iyer V.G., Im W. (2008). CHARMM-GUI: A web-based graphical user interface for CHARMM. J. Comput. Chem..

[48] Abraham M.J., Murtola T., Schulz R., Páll S., Smith J.C., Hess B., Lindahl E. (2015). GROMACS: High performance molecular simulations through multi-level parallelism from laptops to supercomputers. SoftwareX.

[49] Essmann U., Perera L., Berkowitz M.L., Darden T., Lee H., Pedersen L.G. (1995). A smooth particle mesh Ewald method. J. Chem. Phys..

[50] Genheden S., Ryde U. (2015). The MM/PBSA and MM/GBSA methods to estimate ligand-binding affinities. Expert Opin. Drug Discov..

[51] Kumari R., Kumar R., Lynn A. (2014). g_mmpbsa—A GROMACS Tool for High-Throughput MM-PBSA Calculations. J. Chem. Inf. Model..

[52] Miller B.R., McGee T.D., Swails J.M., Homeyer N., Gohlke H., Roitberg A.E. (2012). MMPBSA.py: An Efficient Program for End-State Free Energy Calculations. J. Chem. Theory Comput..

[53] Swanson K., Walther P., Leitz J., Mukherjee S., Wu J.C., Shivnaraine R.V., Zou J. (2024). ADMET-AI: A machine learning ADMET platform for evaluation of large-scale chemical libraries. Bioinformatics.

[54] CLSI (2024). Methods for Dilution Antimicrobial Susceptibility Tests for Bacteria That Grow Aerobically.

