# Supplementary material for: Discovery of Novel Molecular Scaffolds to Overcome Pseudomonas aeruginosa Aminoglycoside Resistance: Insights for a Consensus Scoring Rational Design Approach
Source: Int J Mol Sci. 2026 Mar 13;27(6):2642. doi: 10.3390/ijms27062642 (PMC13026256; doi:10.3390/ijms27062642)
Supplement: Supplementary file 1 [file ijms-27-02642-s001.zip › ijms-4141351-supplementary.pdf]

Discovery of Novel Molecular Scaffolds to Overcome *Pseudomonas aeruginosa* Aminoglycoside Resistance: Insights for a Consensus Scoring Rational Design Approach

Francesco Iesce [1], Jochem Nelen [2], Alejandro Rodríguez-Martínez [2], Carlos Martínez-Cortés [2], Cristina Minnelli [1], Giovanna Mobbili [1], Alessandra Di Gregorio [1], Carla Vignaroli [1], Horacio Pérez-Sánchez [2,*] and Roberta Galeazzi [1,*]

1 Department of Life and Environmental Sciences, Polytechnic University of Marche, via Brecce Bianche, 60131 Ancona, Italy; s1116398@studenti.univpm.it (F.I.); c.minnelli@staff.univpm.it (C.M.)
2 Structural Bioinformatics and High Performance Computing Research Group (BIO-HPC), HiTech Innovation Hub, UCAM Universidad Católica de Murcia, 30107 Murcia, Spain
* Correspondence: hperez@ucam.edu (H.P.-S.); r.galeazzi@staff.univpm.it (R.G.)

Table S1. Gibbs Free binding energy ($\Delta G_{MM/PBSA}$, in kcal/mol) for each first mode selected compound, including their respective standard deviations. BED energy is also reported for purpose of comparison. In bold the experimentally tested compounds.

1st mode	
**Alias code**	**Free energy calculation** +/- Standard Deviation (SD) (kcal/mol)
L1B	**-29.70** ± 4.02
L2B	**-26.40** ± 3.76
**L3B**	**-37.66** ± 3.89
L4B	**-24.67** ± 3.53
L5B	**-26.51** ± 3.16
L6B	**-24.90** ± 3.80
L7B	**-21.13** ± 3.24
L8B	**-24.20** ± 3.46
L9B	**-17.44** ± 3.45
**BED**	**-30.78** ± 3.42

Table S2. Gibbs Free binding energy ($\Delta G_{MM/PBSA}$, in kcal/mol) for each second mode selected compounds, including their respective standard deviations. BED energy is also reported for purpose of comparison. In bold, the experimentally tested compounds.

2nd mode	
**Alias code**	**Free energy calculation**<br>**+/-** Standard Deviation (kcal/mol)
S1B	**-26.54** ± 4.19
S2B	**-26.43** ± 4.26
S3B	**-26.06** ± 3.95
S4B	**-29.08** ± 3.93
S5B	**-27.48** ± 4.26
S6B	**-18.44** ± 4.22
**S7B**	**-33.71** ± 5.32
S8B	**-25.05** ± 4.01
**S9B**	**-35.29** ± 3.09
S10	**-24.27** ± 3.52
S11	**-20.45** ± 4.06
S12	**-27.32** ± 4.59
S13	**-28.47** ± 4.24
S14	**-28.28** ± 4.35
**S15**	**-30.65** ± 3.40
S16	**-19.64** ± 3.67
S17	**-30.22** ± 3.25
S18	**-27.68** ± 4.43
S19	**-16.89** ± 5.93
S20	**-11.68** ± 4.87
**S21**	**-38.17** ± 4.89
S22	**-21.65** ± 3.16
S23	**-18.23** ± 3.78
**S24**	**-26.59** ± 4.98

S25	**-20.82** ± 4.57
S26	**-26.59** ± 5.64
**S27**	**-21.09** ± 3.72
**BED**	**-30.78** ± 3.42

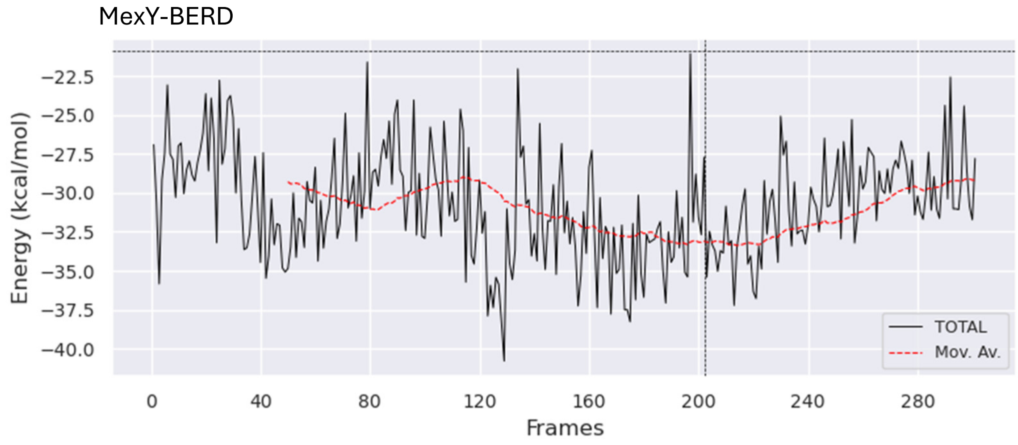

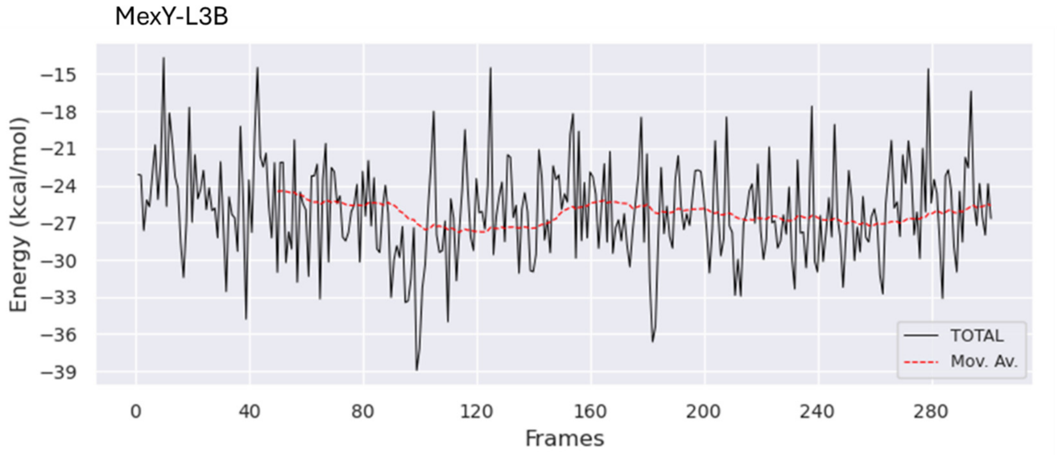

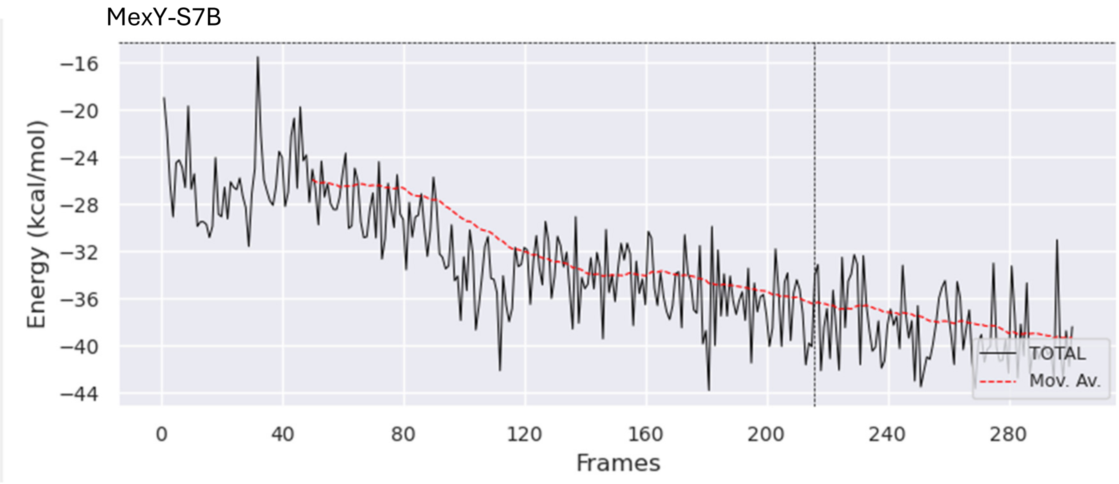

MexY-S9B

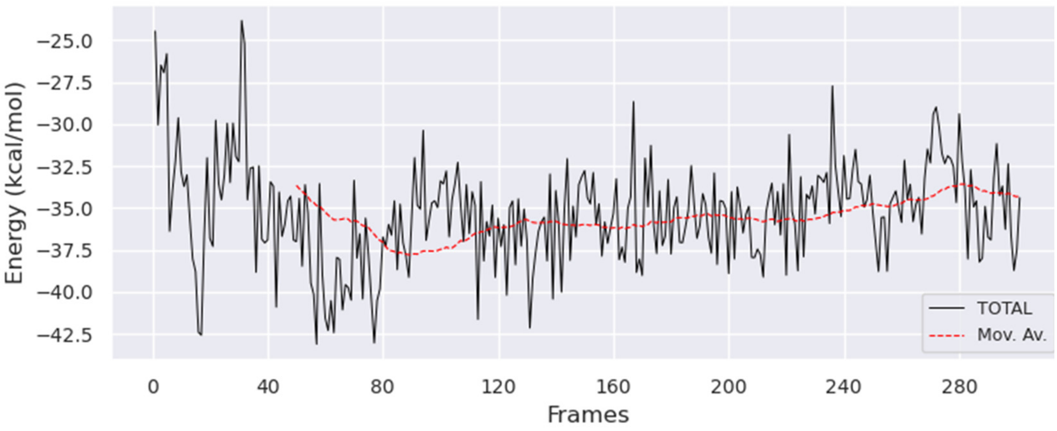

MexY-S15

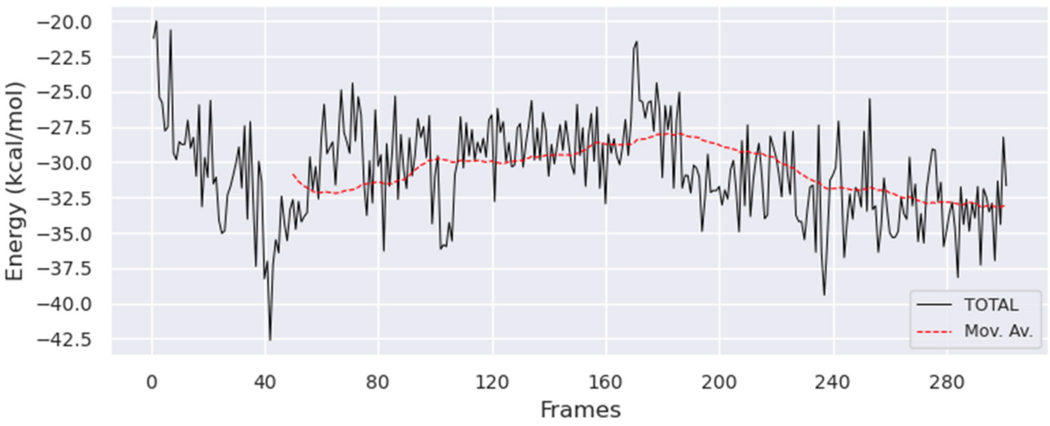

MexY-S21

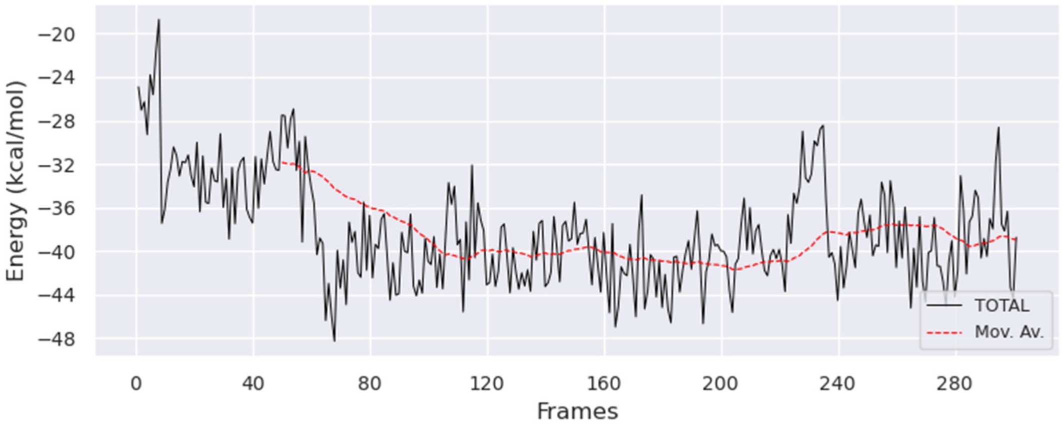

MexY-S24

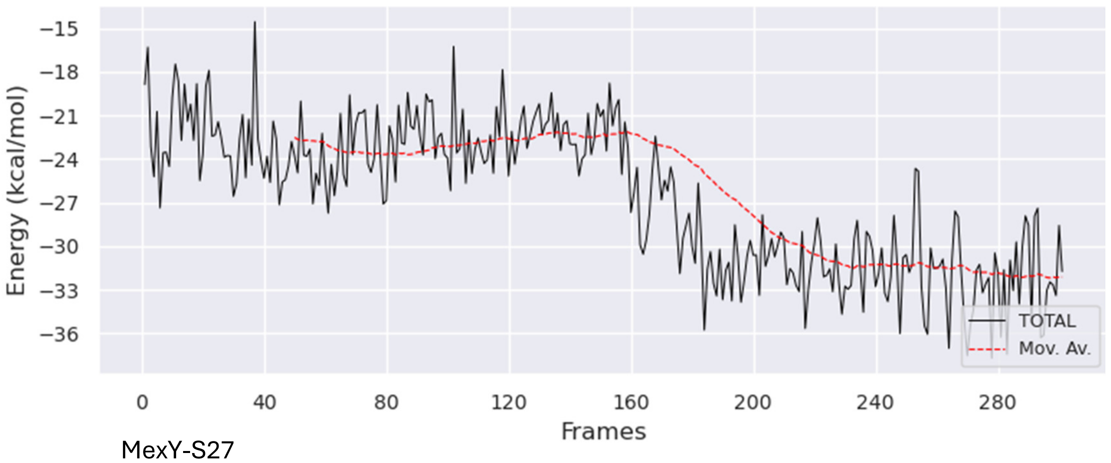

MexY-S27

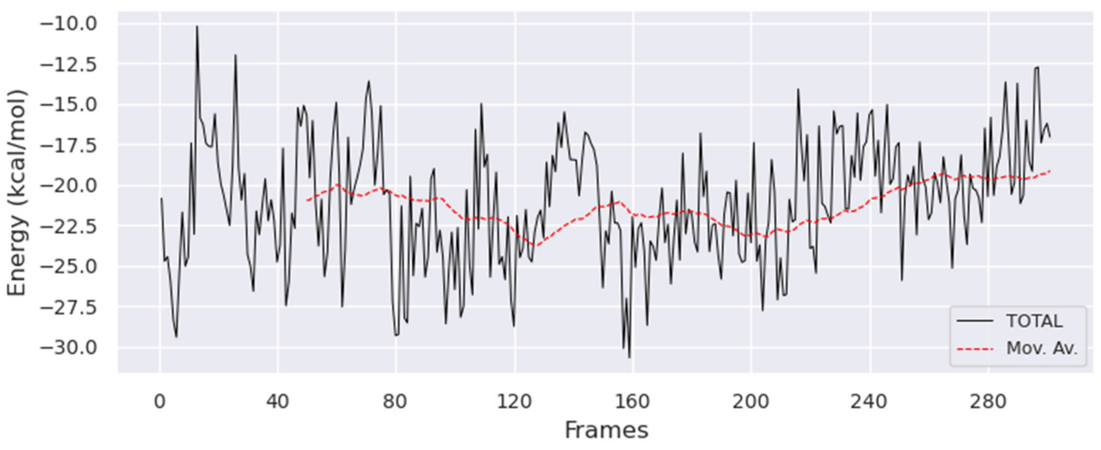

Figure S1. Gibbs free binding energy graphs (MM/PBSA, in kcal/mol) for best performing and tested compounds along the MD trajectory (in frames, 10 frames -1 ns). BED is included as reference compounds.

Microbiological Methods

All *P. aeruginosa* strains were cultured in Luria-Bertani media or on selective *Pseudomonas* agar; Muller Hinton Broth II (MHII) was used for susceptibility assays. All culture media were purchased by Liofilchem S.r.l. (Roseto degli Abruzzi, Teramo, Italy), prepared according to the manufacturer's instructions, and sterilized by autoclaving at 121 °C for 15 minutes. Tobramycin was supplied by Sigma Aldrich (Saint Louis, Missouri, USA). Selected EPI candidates, purchased from Chemspace (https://chem-space.com/) CHEMSPACE LLC. Kyiv, Ukraine, were stored as stock solutions in di-methyl sulfoxide (DMSO) at a concentration of 20 mg/ml and used to determine the minimum inhibitory concentration (MIC) for the reference *Pseudomonas aeruginosa* PA7 and PAO1 strains, using the broth microdilution method in 96-well microtiter plates, following CLSI guidelines [1]. EPI compounds were tested at scalar concentrations ranging from 0.08 to 320 µg/ml, while tobramycin was tested in the range of 0.5-512 µg/ml. *P. aeruginosa* ATCC 27853 was used as the quality-control strain. Checkerboard assays were performed in 96-well microtiter plates by testing scalar concentrations of tobramycin (0.5-512 µg/ml) in combination with EPI compounds (0.625-320 µg/ml).

Table S3. MIC (µg/ml) of tobramycin in association with compounds for *P. aeruginosa* PA7 and PAO1 strains.

	***P .aeruginosa* PA7**							***P. aeruginosa* PAO1**						
	**Tobramycin MIC (µg/ml) with:**							**Tobramycin MIC (µg/ml) with:**						
**Compound concentration** (µg/ml)	**S21**	**S24**	**S27**	**L3B**	**S7B**	**S15**	**S9B**	**S21**	**S24**	**S27**	**L3B**	**S7B**	**S15**	**S9B**
320	NT	NT	NT	NT	NT	128	NT	NT	NT	NT	NT	NT	NT	NT
160	128	128	NT	NT	NT	128	256	NT	NT	NT	NT	NT	NT	NT
80	128	128	256	128	256	128	256	0.5	0.5	NT	0.5	NT	0.5	NT
40	128	128	256	128	256	128	256	0.25	0.25	0.25	0.5	0.5	0.25	0.5
20	128	128	256	128	256	128	256	0.25	0.25	0.25	0.5	0.25	0.25	0.5
10	128	128	NT	128	256	128	256	0.25	0.25	0.25	0.25	0.25	0.25	0.5
5	128	128	NT	128	256	NT	256	0.25	0.25	0.25	0.25	0.25	0.25	0.25
2.5	NT	NT	NT	128	256	NT	256	0.25	0.25	0.25	0.25	0.25	0.25	0.25
1.25	NT	NT	NT	128	256	NT	NT	0.25	0.25	0.25	0.25	0.25	0.25	0.25
0.6	NT	NT	NT	NT	NT	NT	NT	NT	NT	0.25	NT	0.25	NT	0.25
0	256	256	256	256	256	256	256	0.5	0.5	0.5	0.5	0.5	0.5	0.5

NT: not tested concentration due to poor solubility.

Table S4. Conversion Table of first-Mode Compounds and Their Corresponding PubChem Codes.

1st mode	
**Original ID**	**Alias**
Z321002556	L1B
Z241772548	L2B
Z31152510	L3B
Z246233674	L4B
Z44846768	L5B
Z216629660	L6B
Z234939434	L7B
Z246224464	L8B
Z230037408	L9B

Table S5. Conversion Table of Second-Mode Compounds and Their Corresponding PubChem Codes.

2nd mode	
**Original ID**	**Alias**
Z25043713	S1B
Z240725574	S2B
Z1703049238	S3B
Z19578708	S4B
Z55214668	S5B
Z775154182	S6B
Z44488916	S7B
Z1481185012	S8B
Z220420070	S9B
Z48972144	S10
Z992422370	S11
Z46072560	S12
Z46072494	S13
Z46072519	S14
Z29856327	S15
Z56864738	S16
Z46072485	S17

Z769586124	S18
Z1185477737	S19
Z19577835	S20
Z321002556	S21
Z775153226	S22
Z165301940	S23
Z56174882	S24
Z19578648	S25
Z1233447476	S26
Z19316370	S27

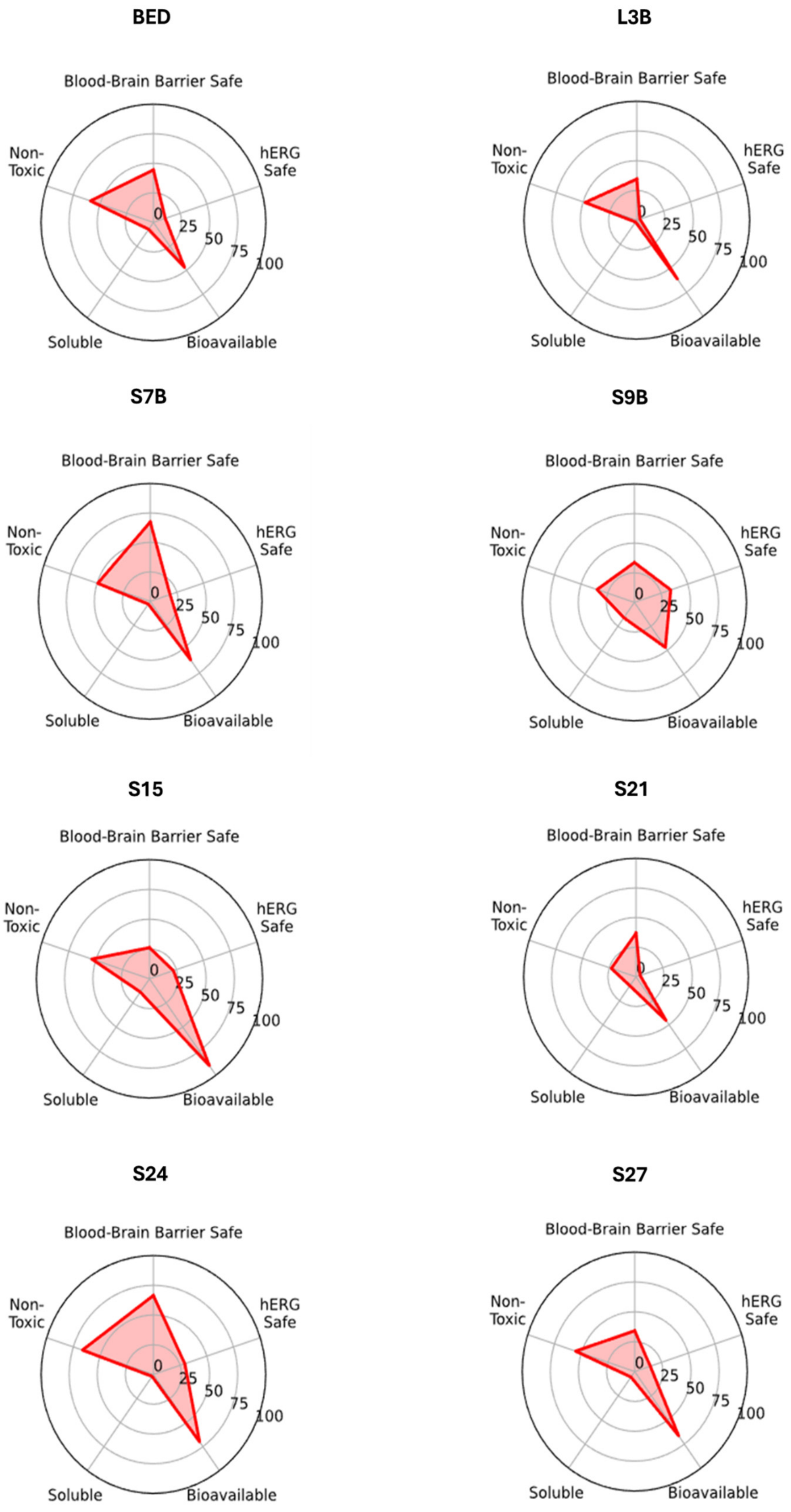

Figure S2. Pharmacokinetic profile evaluations of the selected candidate molecules, including the reference ligand 13-(2-methylbenzyl)-berberine (BED).

Determination of the Octanol/Water Partition Coefficient (logP) for reference compound BED

The octanol/water partition coefficient (logP) was determined using a shake-flask method. Prior to the measurements, n-octanol and water were mutually pre-saturated by vigorous mixing followed by phase separation.The compound (2 mg) was dissolved in 10 mL of pre-saturated n-octanol to obtain the stock solution. An aliquot (5 mL) of this solution was transferred to a vial and mixed with 5 mL of pre-saturated water. The biphasic mixture was agitated for 30 min to allow equilibration between the two phases. The mixture was then centrifuged to ensure complete phase separation.The absorbance of the octanol phase after partitioning was measured at 425 nm using a UV–Vis spectrophotometer (A_final). The absorbance of the initial octanol stock solution was measured at the same wavelength (A_initial).The partition coefficient was calculated according to the following equation:

$$\log P = \log\left(\frac{A_{final}}{A_{initial} - A_{final}}\right)$$

All measurements were performed in triplicate, and the reported value corresponds to the mean of three independent

References

[1] CLSI. *Methods for Dilution Antimicrobial Susceptibility Tests for Bacteria That Grow Aerobically.* 12th ed. CLSI standard M07. Clinical and Laboratory Standards Institute; **2024**.
